# Large-scale deep learning analysis to identify adult patients at risk for combined and common variable immunodeficiencies

**DOI:** 10.1038/s43856-023-00412-8

**Published:** 2023-12-20

**Authors:** Giorgos Papanastasiou, Guang Yang, Dimitris I. Fotiadis, Nikolaos Dikaios, Chengjia Wang, Ahsan Huda, Luba Sobolevsky, Jason Raasch, Elena Perez, Gurinder Sidhu, Donna Palumbo

**Affiliations:** 1 Pfizer Inc, New York, NY USA; 2 National Heart and Lung Institute, Imperial College London, London, UK; 3 Cardiovascular Research Centre, Royal Brompton Hospital, London, UK; 4 School of Biomedical Engineering & Imaging Sciences, King’s College London, London, UK; 5 Department of Biomedical Research, Institute of Molecular Biology and Biotechnology, FORTH, Ioannina, Greece; 6 Unit of Medical Technology and Intelligent Information Systems, University of Ioannina, Ioannina, Greece; 7 Mathematics Research Center, Academy of Athens, Athens, Greece; 8 School of Mathematical and Computer Sciences, Heriot Watt, Edinburgh, UK; 9 Edinburgh Centre for Robotics, Edinburgh, UK; 10 Immunoglobulin National Society, Woodland Hills, CA USA; 11 Midwest Immunology Clinic, Plymouth, MN USA; 12 Allergy Associates of the Palm Beaches, North Palm Beach, FL USA

**Keywords:** Immunological deficiency syndromes, Genetic testing

## Abstract

**Background:**

Primary immunodeficiency (PI) is a group of heterogeneous disorders resulting from immune system defects. Over 70% of PI is undiagnosed, leading to increased mortality, co-morbidity and healthcare costs. Among PI disorders, combined immunodeficiencies (CID) are characterized by complex immune defects. Common variable immunodeficiency (CVID) is among the most common types of PI. In light of available treatments, it is critical to identify adult patients at risk for CID and CVID, before the development of serious morbidity and mortality.

**Methods:**

We developed a deep learning-based method (named “TabMLPNet”) to analyze clinical history from nationally representative medical claims from electronic health records (Optum® data, covering all US), evaluated in the setting of identifying CID/CVID in adults. Further, we revealed the most important CID/CVID-associated antecedent phenotype combinations. Four large cohorts were generated: a total of 47,660 PI cases and (1:1 matched) controls.

**Results:**

The sensitivity/specificity of TabMLPNet modeling ranges from 0.82-0.88/0.82-0.85 across cohorts. Distinctive combinations of antecedent phenotypes associated with CID/CVID are identified, consisting of respiratory infections/conditions, genetic anomalies, cardiac defects, autoimmune diseases, blood disorders and malignancies, which can possibly be useful to systematize the identification of CID and CVID.

**Conclusions:**

We demonstrated an accurate method in terms of CID and CVID detection evaluated on large-scale medical claims data. Our predictive scheme can potentially lead to the development of new clinical insights and expanded guidelines for identification of adult patients at risk for CID and CVID as well as be used to improve patient outcomes on population level.

## Plain language summary

Primary immunodeficiencies (PI) are disorders that weaken the immune system, increasing the incident of life-threatening infections, organ damage and the development of cancer and autoimmune diseases. Although PI is estimated to affect 1-2% of the global population, 70-90% of these patients remain undiagnosed. Many patients are diagnosed during adulthood, after other serious diseases have already developed. We developed a computational method to analyze the clinical history from a large group of people with and without PI. We focused on combined (CID) and common variable immunodeficiency (CVID), which are among the least studied and most common PI subtypes, respectively. We could identify people with CID or CVID and combinations of diseases and symptoms which could make it easier to identify CID or CVID. Our method could be used to more readily identify adults at risk of CID or CVID, enabling treatment to start earlier and their long-term health to be improved.

## Abstract (web summary)

Papanastasiou et al. develop a deep learning-based method to identify combined immunodeficiencies (CID) and common variable immunodeficiencies (CVID) from large-scale electronic health record data. Distinctive combinations of antecedent phenotypes associated with CID/CVID are identified that could improve early diagnosis.

## Introduction

Primary immunodeficiency (PI) is an heterogeneous group of disorders resulting from defects of one or more components of the immune system^1^. PI patients are susceptible to serious, life-threatening infections, organ damage, secondary malignancies and autoimmune diseases^2^. As of 2020, more than 450 PI subtypes have been discovered which were linked to 485 genetic defects^3,4^. This is an increase from over 350 PIs in 2017^5^. As further PI research is conducted, it is anticipated that this number will continue to increase. Early PI diagnosis is critical to improving health outcomes and reducing morbidity and mortality^6^. Improvements in genetic, immunologic, imaging and medical assessments allow the characterization and therapeutic intervention of many PI disorders^4,6^. An important challenge to the early PI diagnosis is the highly heterogeneous clinical presentation, across and within PI subtypes^3–6^. Another critical barrier is the low awareness of PI among primary care practitioners and hence, a lack of referral to clinical immunologists, leading to suboptimal diagnostic evaluation^1,6^.

Awareness campaigns by advocacy groups have identified the warning signs to help identify PI patients (Supplementary Table 1)^7^. Although clinically relevant, this specific set of manifestations does not provide a comprehensive list of clinical phenotypes for systematizing PI screening^8^. Apart from severe combined immunodeficiency (SCID) for which newborn screening is established in the United States, population-based screening for PI does not exist^1–5,9^. Therefore, underdiagnosis, misdiagnosis, or diagnosis delay is common in PI^9,10^. Undiagnosed patients are subject to increased mortality and morbidity^3,4,6^, and are associated with increased healthcare visits and costs^9^. Of note, the National Institute of Health estimates that PI may be affecting 1- 2% of the global population, with recent meta-analyses suggesting that 70-90% of PI patients remain undiagnosed even in countries with well-established diagnostic facilities^10–12^.

Among primary immunodeficiencies, combined immunodeficiencies (CID) are a group of genetic disorders characterized by T-cell impairments, leading to concurrent B-cell and in some cases NK-cell defects^1,9^. The most severe CID subtype, SCID, is characterized by a profound T-cell deficiency^1,3–5,9^. If not treated at early infancy, SCID is fatal. Newborn screening and hematopoietic stem cell or bone marrow transplantation have been established as the gold-standard in treating SCID^9,13^. Other CID subtypes marked by partial but not complete T-cell dysfunction, are associated with variable co-morbidities, and disease progression, and are among the least investigated immune deficiencies^10,13,14^. Unlike SCID, CID patients commonly present with late disease/ symptom onset (>1-year of age) due to residual T-cell function and have a variable clinical presentation (depending upon the individual), hence, their diagnosis cannot be based upon SCID-specific newborn screening^1,13^. Moreover, although a childhood onset is expected due to being genetically-driven, many CVID and CID patients are diagnosed in adulthood due to a lack of awareness hindering childhood diagnosis and/ or delayed disease onset^1,14^. Pneumonia has been shown to be the most frequent severe infection in CID patients^1,13^. CVID is characterized by B-cell defects and is the second most frequent PI (after selective IgA deficiency)^1,2,10^. Currently, CID and CVID have well-established, available treatment options. HSCT and BMT are the clinical standard definitive treatments for CID^1,10^. Immunoglobulin (Ig) replacement therapy is a critical therapeutic intervention in CID and CVID that reduces severe infections, end-organ damage, hospitalizations and overall morbidity and mortality. Concomitant antimicrobial therapies are frequently employed to reduce the severity of infections^13,14^. Considering the underdiagnosis and the available treatments for CID/CVID, it is important to establish methodologies for screening their clinical phenotypes for which there are no other means of systematic identification.

Numerous recent studies demonstrated the merits of machine learning for the accurate analysis of medical claims and electronic health records (EHRs)^15–18^. To enhance the learning process of heterogeneous and sparse features, we developed a deep learning method in which a generalized linear model was incorporated (named as “wide” component thereafter), by adopting a wide and deep learning technique (see details in Supplementary Fig. 1 and 2)^19,20^.

Our study objectives were two-fold. First, we developed and evaluated an accurate deep learning model to analyze administrative medical claims data from nationally representative EHRs (covering all US) towards systematizing the identification of adult patients at risk for CID and CVID. Second, we revealed the most important CID- and CVID-associated clinical phenotypes and their combinations, demonstrating a systematic methodology to potentially improve the identification of adult patients at risk for CID and CVID. Distinctive combinations of antecedent phenotypes associated with CID/CVID were identified, consisting of respiratory infections/conditions (in all Cohorts), genetic anomalies (all Cohorts), cardiac defects (Cohorts 3-4), autoimmune diseases (all Cohorts), blood disorders (Cohorts 1–3) and malignancies (Cohorts 2 and 4), which can possibly be useful to systematize the identification of CID and CVID. The top combinations consisting of antecedent phenotypes with a median of first diagnosis at least 6 months before PI diagnosis were: disorders involving the immune mechanism + decreased white blood cell count + asthma (Cohort 1); non-Hodgkin lymphoma + pneumonia + fever of unknown origin (Cohort 2); bone marrow/stem cell transplant + disorders involving the immune mechanism + asthma (Cohort 3); psoriatic arthropathy + autoimmune disease not elsewhere classified + asthma (Cohort 4).

## Methods

### Data extraction and curation

**Fig. 1:**
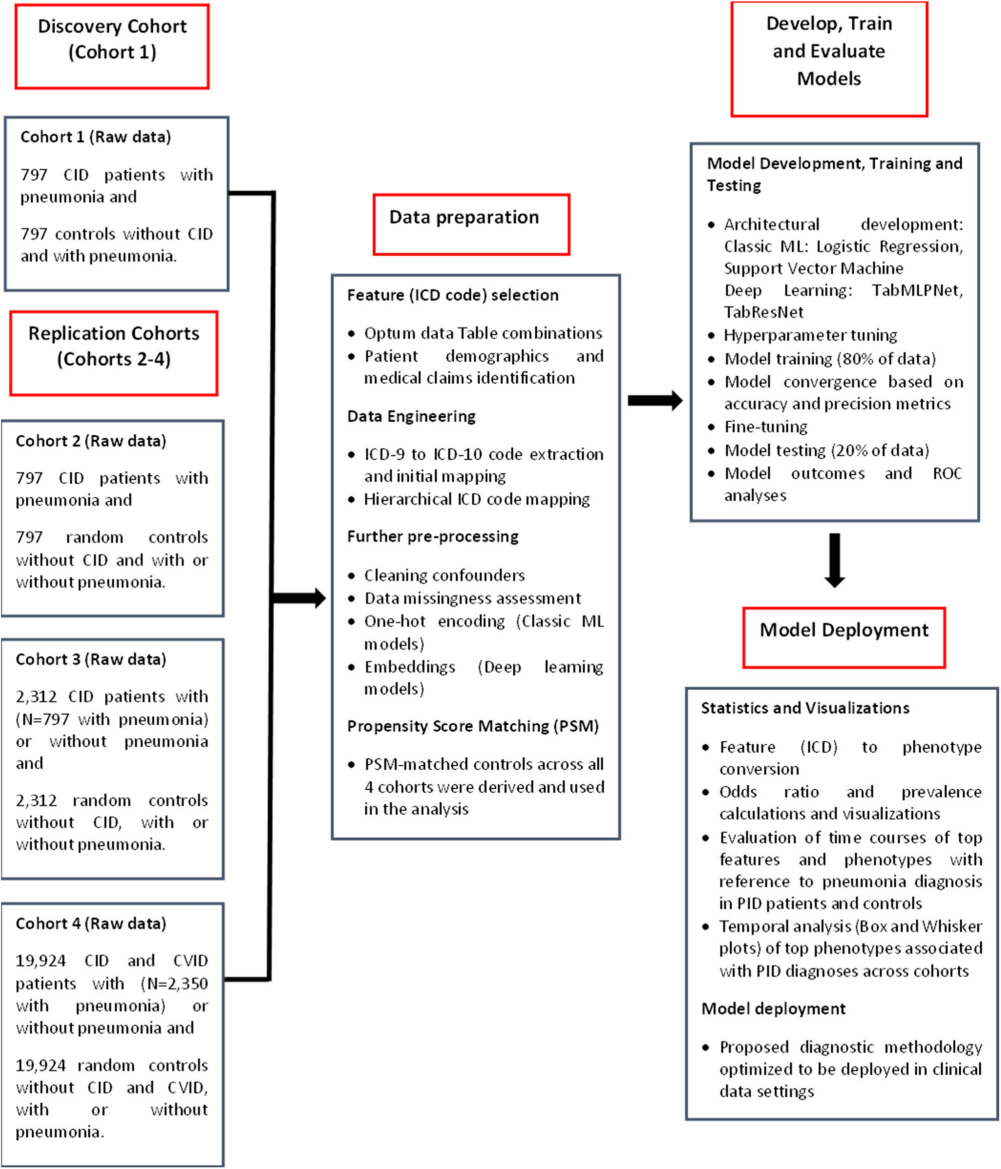
Study workflow. CID Combined immunodeficiency, CVID Common variable immunodeficiency.

All medical claims data used for training (80%) and testing (20%) the machine learning models, were extracted from the Optum® de-identified EHR data (Optum, Inc., Eden Prairie, MN), which is a US nationally representative cohort covering all States. Our observation time frame was from 1 January 2008 to 31 December 2021, which consisted of ~100 million USA patients in total. The data were composed of medical claims containing clinical history and demographics (the latter used for matching; see propensity score matching details in the next paragraph) across all participants. Four large cohorts were generated: 797, 797, 2,312, and 19,924 PI cases and equal control sizes in Cohorts 1-4, respectively (Fig. 1). This makes a total of 47,660 cases and controls. Participants were only included if they were ≥ 18 years old at the time of their PI diagnosis. International Classification of Diseases (ICD) codes for CID and CVID identification were initially derived from the https://www.icd10data.com/. Specifically, all ICD codes for CID and CVID identification were defined as listed in the icd10data D81 and icd10data D83, by considering all main D81 (for CID) and D83 (CVID) sections and subsections. All ICD codes were subsequently confirmed by entering the D81 and D83 sections in the https://icd10cmtool.cdc.gov/ website search engine. The Supplementary Table 2 presents all ICD codes identified in the Optum database for CID/ CVID across Cohorts 1-4 at the time of programming our data extraction.

Across all cohorts, the PI cases and controls were 1:1 matched for age, gender, race, ethnicity, duration of medical history (in months), and the number of healthcare visits, using propensity score (PS) matching (Table 1). This led to an equal number of PI patients and PS-matched controls across cohorts. All ICD-10 or ICD-9 codes that were present in the medical claims for each patient were extracted and the corresponding disease description (see details in “Data preparation and feature selection”) was added in the list of clinical history and considered as co-morbidity. For each patient and control across all cohorts, all ICD-10 and ICD-9 codes were available in the Optum® medical claims data and were automatically extracted and considered in the clinical history, by only excluding data confounders when present, as described in the subsection “Data preparation and feature selection”. Before model training, all the ICD-9 codes present in our data were converted to ICD-10 codes using the updated general equivalence mappings (2018 GEMS) from the https://www.cms.gov/ website (Supplementary Data 1). The presence or absence of all ICD codes identified were used as categorical features for training the machine learning models, without considering the ICD temporal sequence per patient. Our methodology for extracting clinical history in terms of ICD codes prior to machine learning, and ICD code to phenotype conversion, has been previously described^21^.

**Table 1:** Baseline demographics and clinical characteristics of the 4 cohorts included in the study.

Characteristics	Cohort 1		Cohort 2		Cohort 3		Cohort 4	
	PI Cases (*n* = 797)	Controls (*n* = 797)	PI Cases (*n* = 797)	Controls (*n* = 797)	PI Cases (*n* = 2312)	Controls (*n* = 2312)	PI Cases (*n* = 19,924)	Controls (*n* = 19,924)
**Age and Gender**								
Age (years)	46 ± 26	46 ± 25	46 ± 26	48 ± 24	44 ± 26	45 ± 24	47 ± 24	46 ± 23
18–30 (%)	16.39	16.32	16.98	17.02	17.12	17.14	13.89	14.01
31–50 (%)	24.55	24.61	23.96	23.91	23.65	23.69	28.98	29.79
51–70 (%)	35.85	35.79	36.05	35.85	34.81	34.79	35.76	36.03
71-max age (%)	23.21	23.28	23.01	23.22	24.43	24.38	21.37	20.17
Male (%)	46.9	46.3	46.6	45.4	44.3	41.7	38.7	37.8
Female (%)	53.1	53.7	53.4	54.6	55.7	58.3	61.3	62.2
**Ethnicity (%)**								
Caucasian	82.4	85.1	83.4	88.1	81.7	84.2	85.3	86.8
African American	8.7	7.3	8.4	6.7	7.9	7.2	5.8	5.8
Asian	1.4	1.5	1.3	0.5	1.7	1.6	1.2	1.1
Other/Unknown	7.5	6.1	7	4.7	8.7	7.1	7.8	6.3
**Patient History**								
Diagnosis History duration (years)^a^	10 (8–13)	12 (10–14)	10 (8–13)	12 (9–14)	9 (6–12)	11 (8–14)	9 (6–12)	11 (8–14)
Number of visits^a^	201 (103–399)	145 (48–415)	206 (105–399)	182 (45–397)	108 (36–250)	73 (17–243.5)	87 (30–206)	64 (16–195)

^a^Median (25th–75th percentile).

*PI* primary immunodeficiency.

The study was performed with the approval of Pfizer Medical Affairs and the Medical Algorithm/ AI Review committee. Data extraction, pre-processing, model training and testing of the Optum data were performed in accordance with the Declaration of Helsinki. The Optum data have been acquired according to the Health Insurance Portability and Accountability Act (HIPAA) Privacy Rule and all data were fully de-identified before licensed by Pfizer.

### Cohort generation

Figure 1 shows the study workflow, for each cohort. Cohort 1 was initially examined (discovery cohort). As we progressively moved from Cohort 2 up to 4, we aimed to evaluate model diagnostic performance by gradually increasing data heterogeneity and diversifying PI and PS-matched control settings within each cohort increment.

Since pneumonia is the most frequent severe infection in CID^1–7^, we first aimed to investigate whether we can identify CID patients with pneumonia against PS-matched controls with no diagnosis of PI and pneumonia (Cohort 1). To define pneumonia in PI groups and controls, all ICD-10/−9 codes referring to pneumonia subtypes were used from the “Influenza and pneumonia” category in icd10data J09-J18. We then replicated the model training by examining if we can identify CID patients with pneumonia against PS-matched random controls with no diagnosis of PI with and without pneumonia (Cohort 2). Model training was subsequently reproduced to detect CID patients against PS-matched random controls, both with and without pneumonia (Cohort 3). Finally, to increase further data heterogeneity and re-investigate whether models can accurately identify PI in diverse patient settings, we aimed to detect CID and CVID patients against PS-matched random controls, both with and without pneumonia (Cohort 4). Across all cohorts, PS-matched controls had no diagnosis of any type of CID, CVID and PI.

### Data preparation and feature selection

For both PI patients and controls in Cohorts 1-4, patient demographics and ICD-10 / ICD-9 codes were extracted from the Optum® patient and diagnosis tables (using Dataiku; https://www.dataiku.com/) and used as input features for model training. To create model features, ICD codes were converted to corresponding disease descriptions (e.g., ICD-10 code for lymphocytopenia is D72.810, which was converted to “lymphocytopenia”). To perform this step, hierarchical ICD code mapping was implemented using the “regexp_replace” SQL function, by sequentially combining information from the Sub Chapter, Major and Short Description levels. These levels correspond to the diagnosis category, name and description respectively, as obtained from the most updated ICD Data R package (http://cran.nexr.com/web/packages/icd/icd.pdf)^21^. In our implementation, we used the 2020 ICD-10-Clinical Modification release to account for new ICD-10 codes.

As in clinical practice a PI patient may be assigned multiple ICD codes corresponding to general or more specific characterization of PI, all other immunodeficiency-related features identified in the clinical history were removed as confounding variables, to avoid biasing model training (Supplementary Table 2). These confounding variables co-occurred with either D81.89 or D81.9 and were (with ICD-10 code in parentheses): “other specified immunodeficiencies” (D84.8), “nonfamilial hypogammaglobulinemia” (D80.1), “immunodeficiency with predominantly antibody defects” (D80.9), “other immunodeficiencies” (D84.89), “immunodeficiency unspecified” (D84.9), “selective deficiency of immunoglobulin G [IgG] subclasses” (D80.3), “selective deficiency of immunoglobulin A [IgA]” (D80.2), “selective deficiency of immunoglobulin M [IgM]” (D80.4), “immunodeficiency with predominantly antibody defects unspecified” (D80.9), “antibody deficiency with near-normal immunoglobulins or with hyperimmunoglobulinemia” (D80.6), and “other immunodeficiencies with predominantly antibody defects” (D80.8).

### Pre-processing

All pre-processing and machine learning model development were developed in Python 3.7 using pandas, numpy, scipy, matplotlib, GridSearchCV, scikit-learn (for classic machine learning baseline models), and PyTorch widedeep (for deep learning models). Following data preparation, the number of features (ICD codes) identified were Cohort 1: 2,188; Cohort 2: 2,154; Cohort 3: 3,522; and Cohort 4: 10,445 features. For each patient within Cohort 1-4, one-hot encoded categorical values were generated based on whether a patient was positive or negative across each diagnosis ICD code (defined as 1 and 0, respectively). Therefore, the feature dimension “d” for each machine learning model in Cohorts 1-4 was: 2,188 × 2; 2,154 × 2; 3,522 × 2; and 10,445 × 2, respectively. For logistic regression (LR) and support vector machine (SVM), the one-hot encoded categorical values across each ICD code were used as inputs. In deep learning models, the one-hot encoded values were converted into binary value embeddings across each ICD code, using the “tab_preprocessor.fit_transform” (PyTorch widedeep library) function.

### Machine learning models

We developed 2 deep learning models (both with wide and deep components):^19,20^ a multi-layer perceptron (MLP)-model based with dense layers and an MLP model with dense layers in which we incorporated a series of ResNet blocks^22^, named TabMLPNet and TabResNet, respectively (Supplementary Fig. 1). The wide and deep components were jointly trained^19^. The wide (linear model) component was used to learn sparse features via cross-product transformations, whilst the deep component (deep neural network) was focusing to learn dense embeddings in the low-dimensional space^19,20^. The pyramidal architecture of the TabMLPNet model involved 6-layers in total: 3 dense layers of 64 neurons, followed by 3 dense layers of 32 neurons. In our experiments, we observed that model accuracy decreased by 3–5%, as 1–4 more layers were added in the 6-layer TabMLPNet structure. The incorporation of ResNet blocks (with skip connections being able to skip a maximum of two layers, as shown in Supplementary Fig. 1) aims to leverage the flexibility of additional residual functions to be learned inside a deep learning model, as inspired by He et al. ^22^ He et al. showed that by introducing residual learning blocks, the degradation problem can be addressed. The degradation problem is defined as follows: as the network depth increases, accuracy gets saturated and then degrades rapidly^22^. The residual learning (ResNet) blocks are additional layers that are capable to push the residual component to zero when this is optimal during training (and perform “identity mapping”)^22^. This gives flexibility in the model, to either exploit or almost eliminate some layer operations during training. In our analysis, we explored whether through deepening the network structure via using a series of ResNet blocks, would eliminate the degradation-type of problem observed and improve the diagnostic performance of deeper (>6-layer) models. Following experimentation, the architecture of the TabResNet model was consisted of 5 dense ResNet blocks followed by an MLP structure with 4 dense layers of 100, 100, 50 and 50 neurons, respectively. In both models, each dense layer was followed by a ReLU activation. The input dimension (size) for each deep learning model was d x b, where b is the batch size. Both models were trained using a batch size of 128 across 200 epochs, with a dropout of 0.1 per dense layer. Joint training of the wide and deep components was performed using the Adam optimizer by empirically selecting a fixed learning rate of 0.001 (default value for Adam).

We also developed 2 baseline models: LR and SVM-based. To optimize and fine-tune both models, the GridSearchCV library was used to automatically identify their most optimum parameters. For LR, the multi-parametric space on which grid search performed was: regularization penalty (L1, L2), inverse of regularization strength (0.01, 0.1, 1, 10, 100) and class weight (balanced, none). For SVM, a radial basis function kernel was used for which the grid search was: inverse of regularization strength (0.01, 0.1, 1, 10, 100) on L2^2^ regularization and kernel coefficient (0.001, 0.01, 0.1, 1). A cross-validation of 10 and a train-to-test split ratio of 80:20 stratified based on deriving equal numbers of PI and controls in the train and test sets within each cohort were used for all deep and machine (baseline) learning models.

### Mapping ICD codes into phenotypes

Following machine learning model training and testing, we identified ICD codes that were associated with PI diagnoses in Cohorts 1-4. In the context of interpreting the clinical meaning of these features, we converted features into clinical phenotypes (diseases), by using the phenome-wide associations studies (PheWAS) Phecode v.1.2 (dedicated ICD to phenotype grouping system)^23^.

Based on the PheWAS Phecode v.1.2, one or more ICD codes were classified into a distinct phenotype, for each patient. To perform this conversion precisely, the “regexp_replace” SQL function was used to combine information from the Short and Long Description, Major and Sub Chapter levels (see ‘Long_Short_Major_SubChap_ICD_Des.csv’ file in the Supplementary Data 2), as obtained from the most updated ICD Data R package (http://cran.nexr.com/web/packages/icd/icd.pdf^21^. The phenotype mapping file that we created and used is given as Supplementary Data 3. In Supplementary Data 4, we give access to the code developed for main data transformations, pre-processing and machine learning model fitting.

### Statistical analysis

Statistical analyses were performed in R (R Foundation for statistical computing, Vienna, Austria). PS-matching was performed using the MatchIt library (the “glm” distance measure was used). All machine learning models were evaluated by calculating the area under the receiver-operating-characteristic (ROC) curve (AUC). We report the sensitivity, specificity, positive predictive value, negative predictive value, overall accuracy, and ROC AUC (Table 2).

**Table 2:** Diagnostic performance in the testing set of all machine learning models, across all cohorts analyzed

Patient Cohorts					
Metric		Cohort 1	Cohort 2	Cohort 3	Cohort 4
Sensitivity	**TabMLPNet**	**0.88**	**0.87**	**0.82**	**0.87**
	TabResNet	0.86	0.85	0.80	0.86
	LR	0.86	0.84	0.79	0.85
	SVM	0.86	0.85	0.79	0.80
Specificity	**TabMLPNet**	**0.85**	**0.84**	**0.82**	**0.82**
	TabResNet	0.84	0.83	0.81	0.81
	LR	0.82	0.82	0.80	0.75
	SVM	0.82	0.82	0.80	0.79
PPV	**TabMLPNet**	**0.87**	**0.87**	**0.80**	**0.87**
	TabResNet	0.86	0.86	0.79	0.86
	LR	0.85	0.84	0.79	0.83
	SVM	0.85	0.82	0.79	0.80
NPV	**TabMLPNet**	**0.87**	**0.91**	**0.81**	**0.83**
	TabResNet	0.85	0.86	0.79	0.81
	LR	0.84	0.85	0.76	0.78
	SVM	0.83	0.85	0.78	0.79
Accuracy	**TabMLPNet**	**0.87**	**0.87**	**0.80**	**0.85**
	TabResNet	0.86	0.86	0.79	0.84
	LR	0.85	0.85	0.77	0.80
	SVM	0.85	0.84	0.74	0.80
ROC AUC	**TabMLPNet**	**0.94**	**0.93**	0.88*	0.91*
	TabResNet	0.93	0.92	0.87*	0.90*
	LR	0.92	0.91	0.85	0.88
	SVM	0.92	0.91	0.84	0.87

The TabMLPNet model showed the highest diagnostic performance across all cohorts and is indicated with bold letters. The ROC AUC for TabMLPNet and TabResNet were significantly higher compared to LR and SVM in the largest Cohorts 3 and 4. These statistically significant differences are indicated with *. P values for TabMLPNet against LR and SVM were 0.01 and 0.02 and for TabResNet against LR and SVM were 0.02 and 0.03, respectively. No other significant differences were observed between ROC curves.

Odds ratios (ORs) and significance levels for features and phenotypes were calculated using the glm library. Statistical significance was defined as a two-sided *P* value < 0.05. Temporal analysis of the top clinical phenotypes was performed using Box and Whisker plots. Tableau (Tableau 2021.4, Seattle, USA) was used for temporal analysis visualizations.

### Reporting summary

Further information on research design is available in the Nature Portfolio Reporting Summary linked to this article.

## Results

### Participants

The study involved 3 parts as follows: (1) Deep and machine learning models were trained and tested for the diagnosis of CID patients with pneumonia in a large medical claims dataset (Optum; discovery cohort). (2) All models were validated using 2 more CID cohorts from the same dataset, in which the pneumonia filter was removed from the controls and CID cases/controls, respectively. (3) Models were further validated in the largest and most diversified cohort generated, for the diagnosis of CID and CVID patients. All relevant diagnostic codes are listed in Supplementary Table 2.

Patient demographics are shown in Table 1, which reflects the effectiveness of our PS matching process. Age was similar across cohorts and between PI cases and controls (mean age ranging from 44-48 years). Gender, ethnicity, and patient history were also similar between PI cases and controls. Most patients were female (53.1-62.2%) and Caucasian (82.4-88.1%). The number of healthcare visits was consistently higher in PI cases against controls.

### Machine learning model performance

**Fig. 2:**
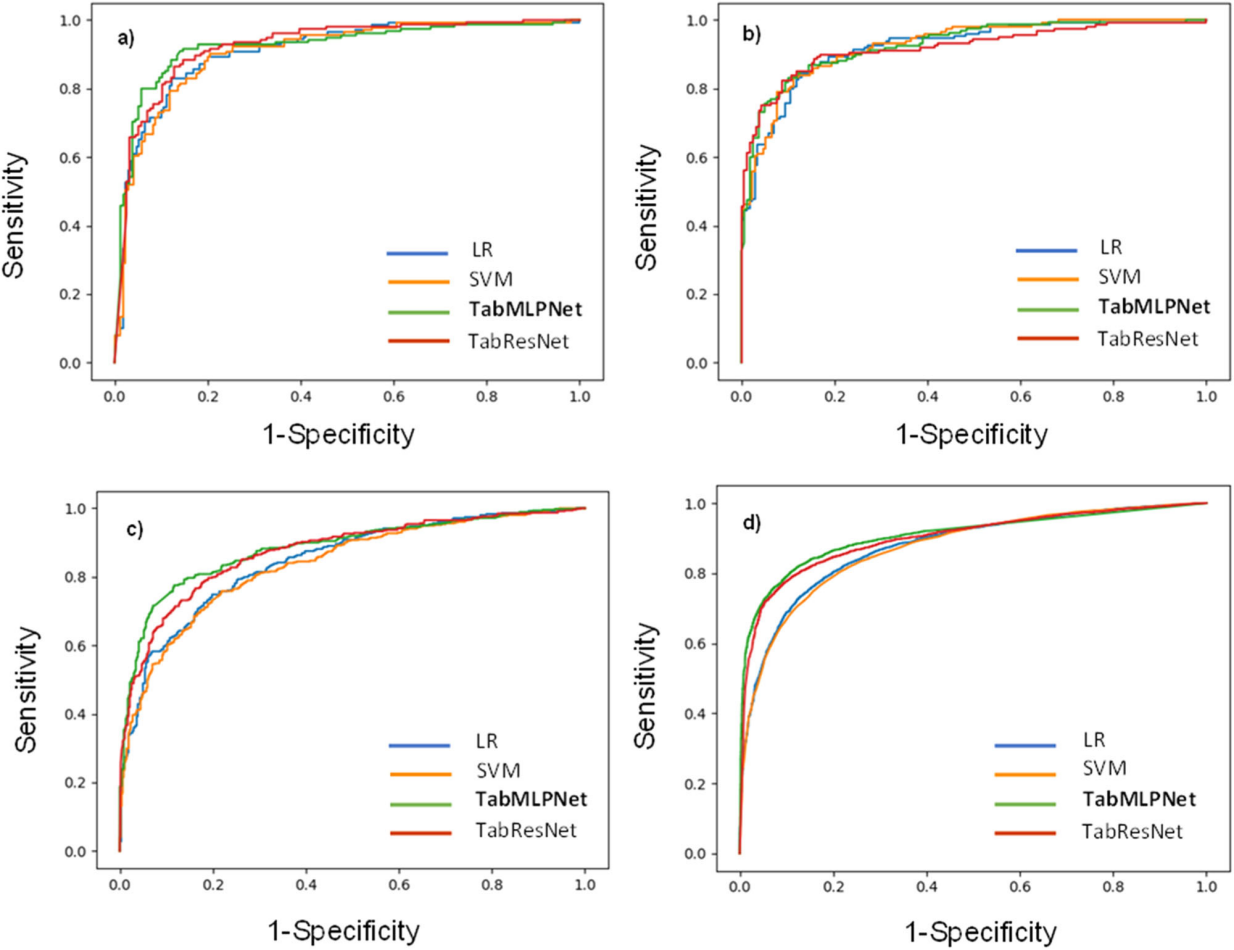
Receiver operating characteristic curves for all machine learning models developed and evaluated in the four cohorts. **a** PI patients with pneumonia against pneumonia patients without PI (*N* = 1594; 797 PI cases and 797 controls). **b** PI patients with pneumonia against randomly selected patients without PI, with and without pneumonia (*N* = 1594; 797 PI cases and 797 controls). **c** PI patients with and without pneumonia against randomly selected patients without PI, with and without pneumonia (*N* = 4624; 2312 PI cases and 2,312 controls). **d** All PI patients (combined and common variable immunodeficiency patients) with and without pneumonia against randomly selected patients without PI, with and without pneumonia (*N* = 39,848; 19,924 PI cases and 19,924 controls). Across all cohorts, PI cases and controls were 1:1 matched for age, gender, race, ethnicity, duration of medical history, and the number of healthcare visits. PI: Primary immunodeficiency.

Initially, we investigated the diagnostic performance of our deep learning models (TabMLPNet, TabResNet) against baseline models (LR, SVM) developed, in identifying PI against PS-matched controls. All model ROC curves are illustrated in Fig. 2.

The TabMLPNet model outperformed all other models across all 4 cohorts, with ROC AUCs ranging from 0.88 to 0.94 (Table 2), showing the highest sensitivity, specificity, positive and negative predictive value, and overall accuracy across all cohorts, ranging from 0.82 to 0.88, 0.82 to 0.85, 0.80 to 0.87, 0.81 to 0.91 and 0.80 to 0.87, respectively.

All other models showed consistently high diagnostic performance in identifying PI against PS-matched controls (ROC AUC range = 0.84-0.93; Table 2). The ROC AUC for TabMLPNet and TabResNet were significantly higher compared to LR and SVM in the largest Cohorts 3 and 4 (Table 2). No other significant differences were observed between ROC curves.

### Assessment of TabMLPNet wide and deep components

Using TabMLPNet, we performed further experiments to examine the performance of the TabMLPNet model with both wide and deep components, against the TabMLPNet model with deep-only and wide-only components.

The TabMLPNet model with wide and deep components showed consistently the highest ROC AUC in identifying PI against PS-matched controls across all 4 cohorts, versus the TabMLPNet model with wide only and deep only components (Supplementary Table 3 and Supplementary Fig. 3). The ROC AUC for TabMLPNet wide and deep were significantly higher compared to TabMLPNet wide only across all cohorts. The ROC AUC for TabMLPNet wide and deep were significantly higher compared to TabMLPNet deep only, in Cohorts 1 and 3.

### Clinical phenotype importance

The second aim of the study was to identify the most important CID- and CVID-associated clinical phenotypes per cohort. Diagnostic codes were converted into clinical phenotypes and their ORs were calculated.

**Fig. 3:**
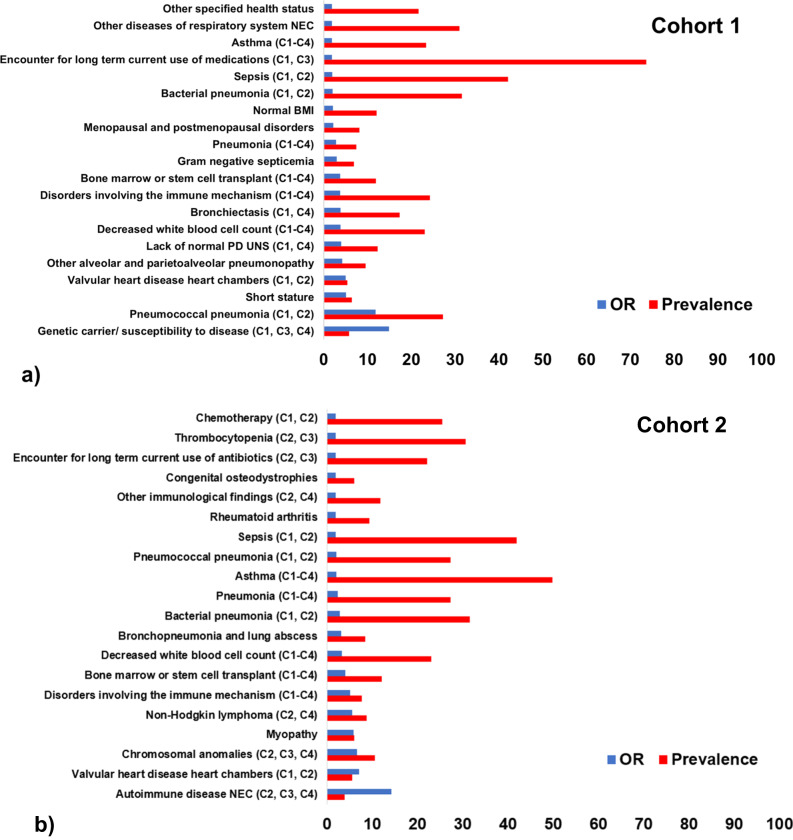
Odds ratio (OR, blue) and prevalence (%, red) for the top clinical phenotypes associated with PI, identified in Cohorts 1-2. **a** Cohort 1 (*N* = 1594); (**b**) Cohort 2 (*N* = 1594). All clinical phenotypes significantly associated with the diagnosis of PI that had an OR > 1.5 were included in the illustrations. Univariate logistic regression was used to calculate the odds ratios. PD: Physiological development, UNS: Unspecified, NEC: Not elsewhere classified, F: Family, P: Personal, CC: Certain conditions, LH: Lymphoid hematopoietic, C1-C4: Declare congruent phenotypes in Cohorts 1-4.

**Fig. 4:**
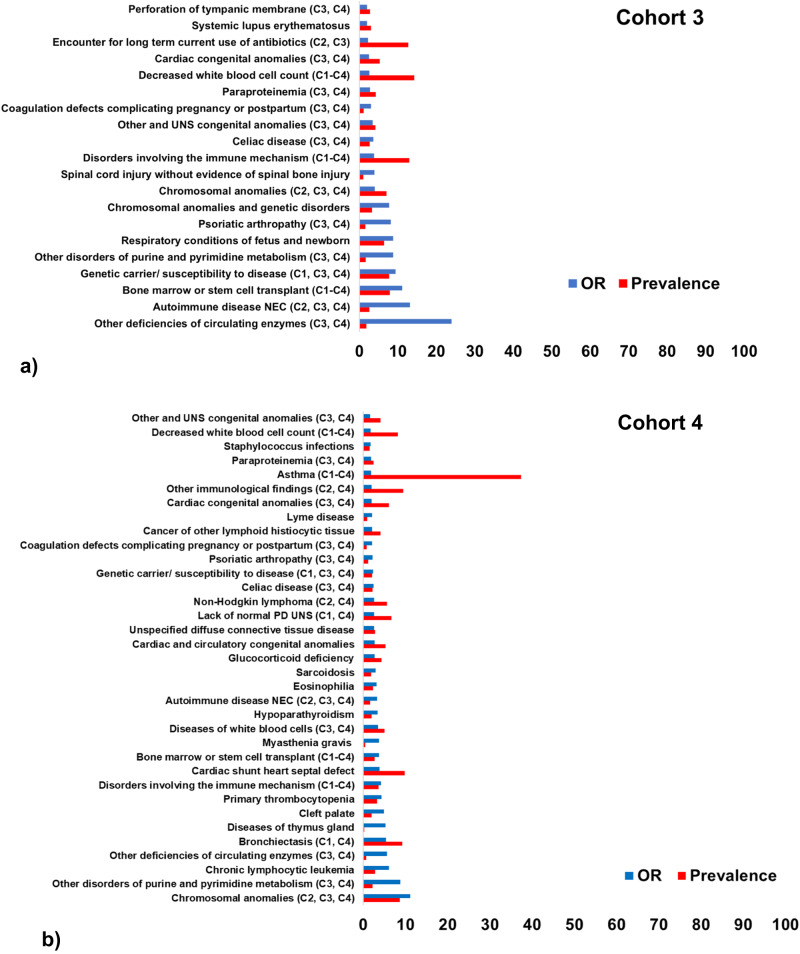
Odds ratio (OR, blue) and prevalence (%, red) for the top clinical phenotypes associated with PI, identified in Cohorts 3-4. **a** Cohort 3 (*N* = 4624); (**b**) Cohort 4 (*N* = 39,848). All clinical phenotypes significantly associated with the diagnosis of PI that had an OR > 1.5 were included in the illustrations. Univariate logistic regression was used to calculate the odds ratios. PD: Physiological development, UNS: Unspecified, NEC: Not elsewhere classified, F: Family, P: Personal, CC: Certain conditions, LH: Lymphoid hematopoietic, C1-C4: Declare congruent phenotypes in Cohorts 1-4.

In Cohorts 1–4, the OR of the top clinical phenotypes ranged from 14.91-1.85, 14.24-1.83, 23.97-1.95, and 11.12-1.66, respectively (Figs. 3 and 4). For Cohorts 1–3, the top twenty phenotypes are presented (Figs. 3a, b and 4a). For the largest Cohort 4, the top 35 phenotypes are shown, reflecting a greater number of phenotypes reaching high statistical significance (Fig. 4b). A full list of all phenotype ORs, prevalence and statistical significance across cohorts is given in the Supplementary Data 5.

Several phenotypes were revealed in Cohorts 1-4 (Figs. 3, 4). In Cohort 1, genetic carrier/susceptibility to disease, pneumococcal pneumonia, short stature, valvular heart disease and alveolar/parietoalveolar pneumonopathy were the 5 strongest phenotypes (Fig. 3a). In Cohort 2, autoimmune disease not-elsewhere-classified (NEC), valvular heart disease, chromosomal anomalies, myopathy and non-Hodgkin lymphoma were the 5 top phenotypes (Fig. 3b). In Cohort 3, deficiencies of circulating enzymes, autoimmune disease NEC, bone marrow/stem cell transplant, genetic carrier/susceptibility to disease and disorders of purine/pyrimidine metabolism were the top phenotypes (Fig. 4a). In the largest Cohort 4, chromosomal anomalies, disorders of purine/pyrimidine metabolism, chronic lymphocytic leukemia, deficiencies of circulating enzymes and bronchiectasis were the strongest phenotypes (Fig. 4b).

Across all cohorts, various other genetic, respiratory, autoimmune, musculoskeletal, blood and blood cancer diseases were revealed (Figs. 3, 4; Supplementary Notes). The ORs of the underlying diagnostic codes across all cohorts were also computed and are presented in the Supplementary Figs. 4-7.

### Temporal distributions

**Fig. 5:**
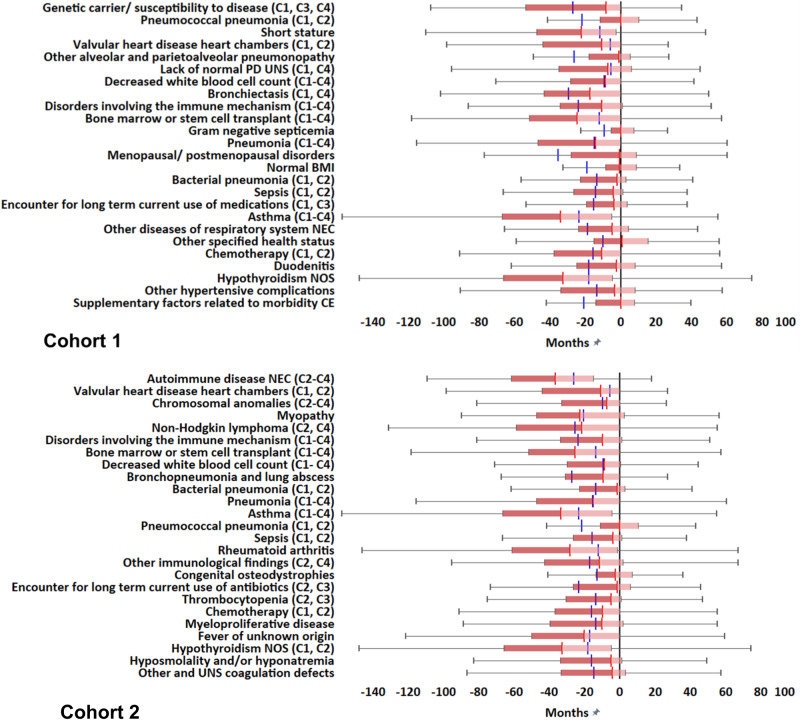
Temporal analysis of the top 25 clinical phenotypes identified in Cohorts 1-2 (by odds ratio). Cohort 1 (*N* = 1,594); Cohort 2 (*N* = 1,594). Temporal analysis was estimated in terms of Box and Whisker plots. The red line within Box and Whisker plots represents the median. The pink distribution indicates the lower interquartile value to the median. The dark red distribution indicates the upper interquartile value to the median. The blue line illustrates the median of the pneumonia diagnosis when present in the data. The Box and Whisker plots are calculated in reference to the PI diagnosis showed as black solid line in each Cohort illustration. C1-C4: Declare congruent phenotypes in Cohorts 1-4. The top 25 clinical phenotypes significantly associated with the diagnosis of PI were included in the illustrations.

**Fig. 6:**
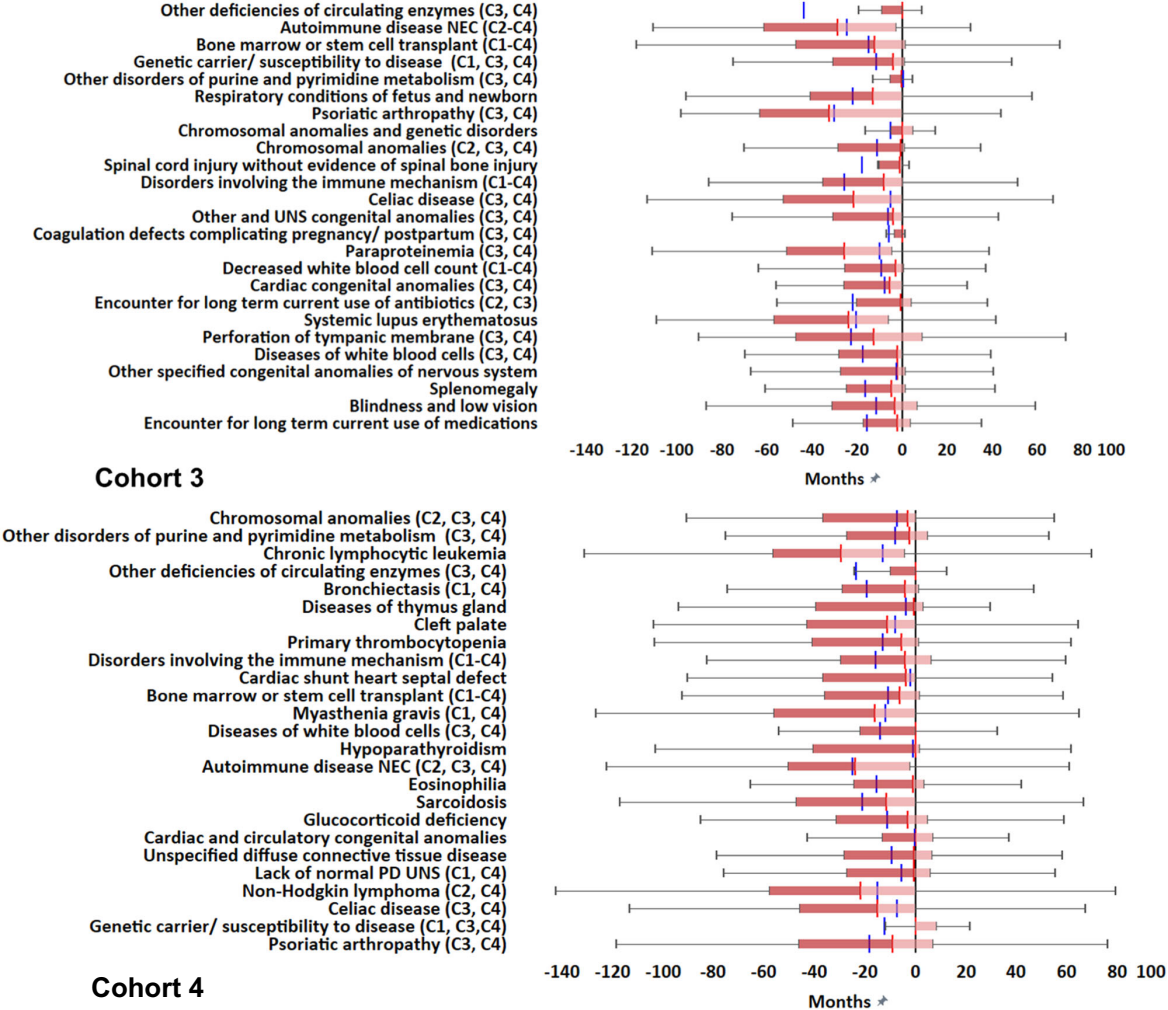
Temporal analysis of the top 25 clinical phenotypes identified in Cohorts 3-4 (by odds ratio). Cohort 3 (*N* = 4,624); Cohort 4 (*N* = 39,848). Temporal analysis was estimated in terms of Box and Whisker plots. The red line within Box and Whisker plots represents the median. The pink distribution indicates the lower interquartile value to the median. The dark red distribution indicates the upper interquartile value to the median. The blue line illustrates the median of the pneumonia diagnosis when present in the data. The Box and Whisker plots are calculated in reference to the PI diagnosis showed as black solid line in each Cohort illustration. C1-C4: Declare congruent phenotypes in Cohorts 1-4. The top 25 clinical phenotypes significantly associated with the diagnosis of PI were included in the illustrations.

We derived the temporal distributions of the 25 most important clinical phenotypes, by tracking the first date of each phenotype diagnosis with reference to PI diagnosis per patient (Figs. 5–6, Supplementary Notes).

Most phenotypes preceded PI diagnoses across all cohorts. The number of phenotypes that had a median of first diagnosis greater than 3 months before PI diagnosis in Cohorts 1-4 were: 16, 21, 15 and 15, respectively. Their median value range in months before PI diagnosis were: 34.1-3.4, 36.5-4.0, 32.4-3.1, and 29.3-3.2, respectively. At a threshold of 6 months before PI diagnosis, the phenotype numbers were 12, 17, 9, and 9 in Cohorts 1-4, respectively.

### Time frame of diagnoses prior to pneumonia

In Cohort 1, 20-year time frames of ICD codes and clinical phenotypes in CID cases against controls were computed, prior to (-10 years) and after (+10 years) the first diagnosis of pneumonia, used as a common feature between cases and controls (Supplementary Figs. 8, 9). The 20-year time frames depict the cumulative proportion of patients with each phenotype, which equals the sum of the proportions from each of the years preceding or following pneumonia diagnosis.

It is obvious that most ICD codes and phenotypes started being diagnosed before the first pneumonia diagnosis in CID cases against controls. All pneumonia subtypes identified in our study (as derived from our largest Cohort 4) are provided in the Supplementary Data 6.

### Combinations

**Fig. 7:**
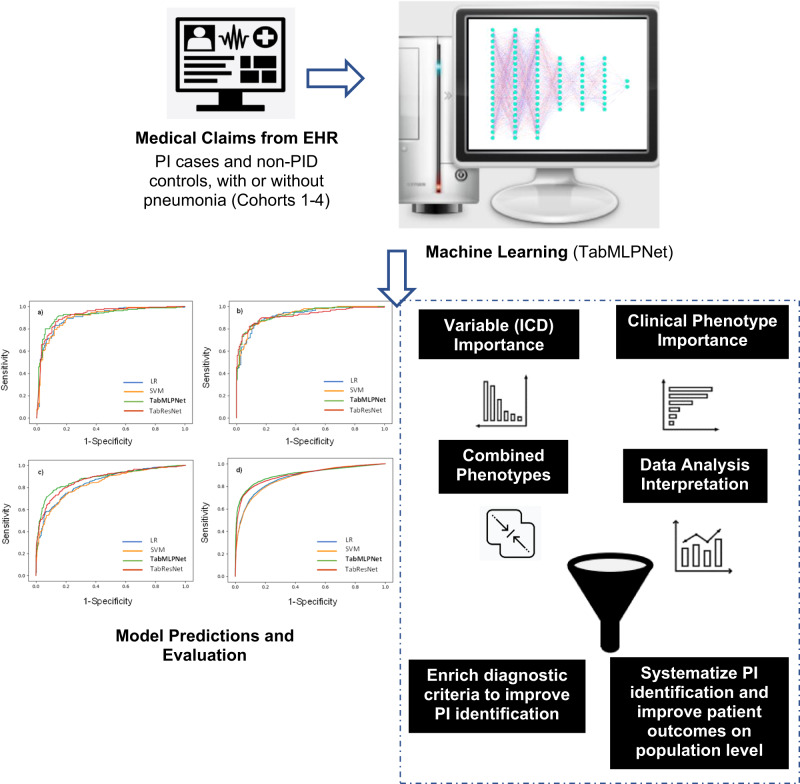
Development and evaluation of a machine learning model pipeline for improving and systematizing the identification of PI (CID and CVID). **a**–**d** diagnostic performance of all models tested across Cohorts 1–4, respectively. US nationally representative medical claims were used to develop a cohort of combined immunodeficiency (CID) patients and non-CID controls (both with pneumonia, Cohort 1). Diagnosis (ICD) codes were extracted and used as variables to train the TabMLPNet model. Subsequently, the same methodology and model were internally tested in 3 replication cohorts (Cohorts 2-4). To derive clinical insights for the identification of patients at risk for PI across all cohorts, the ICD codes were then converted to clinical phenotypes and odds ratios were calculated to estimate hierarchical clinical phenotype importance (Cohorts 1-4). Further, phenotype temporal profiles and combinations were extracted and assessed in terms of their associations with PI. Clinical phenotypes can be used to enrich the diagnostic criteria for the early PI detection, including expanding the existing clinical warning signs and improving patient outcomes on population level. EHR: Electronic Health Records, CID: Combined immunodeficiency, CVID: Common variable immunodeficiency, PI: Primary immunodeficiency.

Further, we estimated ORs of combined phenotypes associated with PI (Table 3, Supplementary Tables 4-5). Various heterogeneous combinations were revealed, mostly consisting of antecedent phenotypes with a median of first diagnosis at least 6 months before PI diagnosis (Table 3, Supplementary Tables 4-5, Figs. 5, 6). An illustration of our entire methodology is shown in Fig. 7.

**Table 3:** Top 5 combinations of clinical phenotypes and their association with PI (Cohorts 1-4).

Phenotype Combination	Odds Ratio (95% CI)	P values	Number of phenotypes
**Cohort 1**			
Pneumococcal pneumonia; Disorders involving the IM;** Asthma.**	5.98 (4.67-7.29)	0.0008	3 / 2 / 2
Other alveolar and parietoalveolar pneumonopathy; Bacterial pneumonia; Asthma.**	6.13 (4.39-7.87)	0.0005	3 / 1 / 1
Valvular heart disease/ heart chambers;** ENC for LT use of MED;* Asthma.**	6.46 (5.68-7.24)	0.0004	3 / 3 / 2
Decreased WBC count;** Bacterial pneumonia; ENC for long term use of MED;* Asthma.**	6.97 (4.89-9.05)	0.0001	4 / 3 / 2
Disorders involving the IM;** Decreased WBC count;** Asthma.**	6.53 (2.22-8.75)	0.0003	3 / 3 / 3
**Cohort 2**			
Non-Hodgkin lymphoma;** Pneumonia;** Fever of unknown origin.**	6.96 (3.76-10.20)	0.0001	3 / 3 / 3
Thrombocytopenia;* Non-Hodgkin lymphoma;** Pneumonia.**	6.75 (3.49-10.01)	0.0001	3 / 3 / 2
Pneumococcal pneumonia; Non-Hodgkin lymphoma;** Fever of unknown origin.**	5.88 (3.74-8.01)	0.0008	3 / 2 / 2
Asthma;** ENC for long term use of antibiotics; Fever of unknown origin.**	6.33 (3.27-9.39)	0.0002	3 / 2 / 2
Myeloproliferative disease;** Asthma;** Fever of unknown origin.**	5.89 (3.23-8.54)	0.0008	3 / 3 / 3
**Cohort 3**			
Bone marrow /stem cell transplant;** Disorders involving the IM;** Asthma.**	6.38 (4.67-8.29)	0.0003	3 / 3 / 3
Pneumonia;** Bone marrow /stem cell transplant;** Disorders involving the IM.**	6.35 (3.87-8.84)	0.0003	3 / 3 / 3
Genetic carrier /susceptibility to disease;* Asthma.**	6.78 (5.02-8.54)	0.0001	2 / 2 / 1
Genetic carrier /susceptibility to disease;* Other disorders of purine and pyrimidine metabolism.	5.81 (4.72-6.89)	0.0008	2 / 1 / 0
Decreased WBC count;* Cardiac congenital anomalies.*	6.83 (4.22-9.44)	0.0001	2 / 2 / 0
**Cohort 4**			
Chromosomal anomalies;* Cardiac congenital anomalies; Lack of normal PD UNS.	6.09 (5.75-6.43)	0.0005	3 / 1 / 0
Disorders involving the IM;* Non-Hodgkin lymphoma;** Asthma.**	6.07 (5.71-6.44)	0.0005	3 / 3 / 2
Bone marrow /stem cell transplant;** Disorders involving the IM.*	5.86 (5.31-6.41)	0.0008	2 / 2 / 1
Bone marrow /stem cell transplant;** Bronchiectasis;* Non-Hodgkin lymphoma.**	5.98 (3.75-8.21)	0.0008	3 / 3 / 2
Psoriatic arthropathy;** Autoimmune disease NEC;** Asthma.**	6.25 (4.73-7.77)	0.0002	3 / 3 / 3

The table presents combinations which had at least one phenotype in addition to PI. Phenotypes were selected hierarchically (based on ORs; see Figs. 3–6), introducing at least one new phenotype combination in each table row (per cohort) and by including the highest number of possible combinations with significant ORs>3.00. The X/ Y/ Z numbering indicates the total number of phenotypes in each combination and how many of these had a median of first diagnosis greater than 3 (indicated with *) and 6 months (**) before PI diagnosis, respectively. *PD* Physiological development, *UNS* Unspecified, *ENC* Encounter, *LT* Long-term, *MED* Medications, *WBC* White blood cell, *IM* Immune mechanism, *NEC* Not elsewhere classified. Note: valvular heart disease/ heart chambers involve all types of valve diseases and undefined cardiomyopathy.

In Cohorts 1-4, the top combinations consisted of antecedent phenotypes with a median of first diagnosis at least 6 months before PI diagnosis were: disorders involving the IM + decreased white blood cell (WBC) count + asthma (OR = 6.53, 95% CI: 2.22–8.75); non-Hodgkin lymphoma + pneumonia + fever of unknown origin (OR = 6.96, 95% CI: 3.76–10.20); bone marrow/stem cell transplant + disorders involving the IM + asthma (OR = 6.83, 95% CI: 4.22–9.44); psoriatic arthropathy + autoimmune disease NEC + asthma (OR = 6.25, 95% CI: 4.73–7.77), respectively.

## Discussion

We have performed a large-scale analysis of medical claims data derived from a nationally representative EHR database (global US, covering all States), by devising a deep learning-based methodology. Our method showed consistently high diagnostic performance in identifying CID/CVID across 4 cohorts with clinically diverse patient profiles. Furthermore, we identified the top antecedent phenotypes associated with these PI. We also revealed the top phenotype combinations for each cohort and showed that they consist mostly of antecedent heterogeneous diseases.

To the best of our knowledge, we were the first to interrogate large medical claims data for the identification of patients at risk for CID/CVID and of antecedent phenotype combinations through deep learning and OR calculations. Our large-scale deep learning method was performed on US representative medical claims, showed high diagnostic performance, presented an extensive statistical / temporal analysis of antecedent phenotypes and phenotype combinations that were associated with CID/CVID, and is therefore transferable to external clinical settings. Our model can also be potentially applied to the identification of other PI disorders. Moreover, none of the previous studies focused on CID^24–27^. The most recent work was a single-center observational study by Mayampurath et al who analyzed 6,422 patients, of whom only 247 had been diagnosed with PI^24^. By modeling co-morbidities (clinical history), their best-performing Random Forest model reached a moderate ROC AUC of 0.65 (95% CI: 0.62-0.68) in identifying PI, which was improved to 0.72 (95% CI: 0.69-0.75) when laboratory results and radiology procedures were considered. The moderate performance in this study can mainly be due to the small PI cohort used, which led to extracting a limited number of phenotypes that were evaluated against PI. Although this study did not explore deep learning methodologies, their classic machine learning models (Random Forest and Logistic Regression) on clinical history, reached considerably lower performance (ROC AUCs: 0.62-0.65) compared to our baseline model results (LR and SVM with ROC AUCs ranging from: 0.84-0.92; Table 2). Rider et al developed a Bayesian network consisted of known risk factors and showed 87% and 91% sensitivity and specificity in discriminating PI against controls, using 3,460 pediatric patients (~50% with PI)^25^. Abyazi et al, identified different proteomic profiles in patients with noninfectious complications against uncomplicated CVID, implementing unsupervised learning in 72 participants^26^. Emmaneel et al., developed a computational pipeline to discriminate CVID from other PI and healthy controls, using flow cytometry data from 179 participants^27^. Unlike our work, the last 2 studies focused on evaluating differences in the molecular profiles of PI patients and did not aim to improve PI identification in the frontline of clinical practice. By improving PI identification via population-based screening, it is possible to substantially reduce morbidity, mortality, healthcare visits and costs, through timely patient access to available definitive and supportive treatments for both CID and CVID. Our method reached high diagnostic performance using large medical claims data and revealed phenotypes and combinations that can possibly have merit for the systematic identification of CID and CVID.

Our findings are clinically important because our predictive scheme detected disease combinations, which are the first-time to be reported for the possible identification of patients at risk for PI^1,2,10,13^. In particular, there were distinctive combinations of antecedent (>6 months) phenotypes such as respiratory infections or conditions (asthma, pneumonia, bronchiectasis), genetic anomalies (genetic carrier/susceptibility to disease, lack of normal PD, chromosomal anomalies), cardiac defects, autoimmune diseases (psoriatic arthropathy, autoimmune disease NEC, celiac disease, disorders involving the immune mechanism), blood disorders and malignancies (non-Hodgkin lymphoma), associated with both CID and CVID (Table 3, Supplementary Tables 4-5, Figs. 5, 6). Since Cohort 4 involves both CID and CVID, the phenotype combinations revealed can possibly increase early suspicion of potential PI before further categorization to CID or CVID. Validating further our proposed method on external medical claims, these respiratory and non-respiratory combinations can potentially help to expand the existing clinical warning signs and to systematize the identification of patients at risk for CID/CVID.

Most individual respiratory, genetic, autoimmune, blood and malignancy phenotypes revealed across cohorts are reported in the literature and recent PI surveys (Figs. 3, 4)^1–3,10–14^. Numerous congruent phenotypes were identified across Cohorts 1-4. Of note, there were also unknown individual phenotypes emerged, such as asthma (in Cohorts 1-4), coagulation defects complicating pregnancy or postpartum (Cohorts 3-4) and cancer of lymphoid histiocytic tissue (Cohort 4)^2,13^. Among the most severe early manifestations, chronic lymphocytic leukemia was the third most important antecedent phenotype in Cohort 4 (Figs. 4 and 6). Despite hematologic malignancies are known to be associated with PI, there is low awareness of chronic lymphocytic leukemia in PI patients^28^. Moreover, hypothyroidism (Cohorts 1-2), autoimmune diseases NEC (Cohorts 2-4), systemic lupus erythematosus (Cohort 3), psoriatic arthropathy (Cohorts 3-4), rheumatoid arthritis (Cohort 2) and celiac disease (Cohorts 3-4) were the top antecedent autoimmune conditions associated with CID/CVID. Our findings can therefore potentially raise awareness and support treatment optimization strategies for co-treating early both the underlying disorder (CID/CVID) and each of these respiratory, oncological and endocrinological diseases.

As described, among the most important phenotypes on OR analysis, several antecedent CID and CVID-associated phenotypes corresponding to autoimmune diseases have been identified across cohorts. We refer to these phenotypes together with the cohort(s) in which each phenotype was identified (from Figs. 3, 4) and median values in months prior to the first PI diagnosis (from Figs. 5, 6): hypothyroidism (in Cohorts 1 and 2; median values = -32.7 and -34.2 months respectively prior to PI diagnosis), disorders Involving the immune mechanism (Cohorts 1-4; median values = −13.6, −10.8, −9.4 and −8.9 months respectively), autoimmune disease not elsewhere classified (Cohorts 2-4; median values = −36.5, −28.8, and −23.7 months respectively), rheumatoid arthritis (Cohort 2; median value = −16.3 months), psoriatic arthropathy (Cohorts 3 and 4; median values = −32.4 and −10.8 months respectively), celiac disease (Cohorts 3 and 4; median values = −23.9 and −16.9 months respectively), systemic lupus erythematosus (Cohort 3; median value = −25.9 months) and sarcoidosis (Cohort 4; median value = −15.8 months). These findings clearly show that the diagnoses of autoimmune diseases have consistently preceded PI diagnoses. Αs also mentioned above, antecedent CID/ CVID-associated phenotypes corresponding to malignancies were also observed, such as non-Hodgkin lymphoma (Cohorts 2 and 4; median values = −21.5 and −21.7 months, respectively) and chronic lymphocytic leukemia (Cohort 4; median value = − 29.3 months). In addition, bone marrow/ stem cell transplant (Cohorts 1-4; median values = -24.6, −27.3, −15.5 and −8.2 months, respectively) and chemotherapy (Cohorts 1 and 2; median values = −16.9 and −13.8 months respectively) were among the most important antecedent phenotypes. It is known that bone marrow/ stem cell transplantation following high-dose chemotherapy is increasingly used for the treatment of autoimmune disease and chronic lymphocytic leukemia patients, not sufficiently responding to conventional treatments^29–31^. Chemotherapy-based therapies are the standard of care treatments for non-Hodgkin lymphoma and chronic lymphocytic leukemia^31^. Demonstrating that autoimmune diseases and malignancies have consistently preceded PI diagnoses, can explain the prevalence of bone marrow/ stem cell transplant and chemotherapy phenotypes in our analysis. It is known that autoimmune diseases, blood malignancies, bone marrow/ stem cell transplant and/ or chemotherapy can induce secondary immunodeficiency (SI, through mainly B-cell dysfunction in some patients)^3,4,32^. Although there may be challenges in differentiating PI from SI (especially when autoimmune disease or blood malignancy treatment precedes diagnostic testing for PI), current evidence shows that some antibody deficiencies initially attributed to SI may instead be due to an underlying PI^32^. These findings indicate the need for long-term administration of Ig replacement therapy in these patients^32^. Independently of PI and SI crossovers in patients with autoimmune disease/ blood malignancy, our method can be possibly useful for improving the identification of patients at risk for PI, before further characterization, monitoring and treatment of immunodeficiency by expert immunologists. Therefore, our large-scale analysis can be potentially applicable to systematize CID/ CVID identification and improve patient outcomes.

Table 1 shows the effectiveness of our PS matching process between PI cases and controls. Age, gender, ethnicity, and patient history were similar between cases and controls. Consistent with clinical experience reported in recent surveys^1,2,10^, the number of visits were consistently higher in PI cases against controls.

CID is characterized by complex immune defects and are among the least investigated PI^10,13,14^. Since pneumonia is their most frequent severe infection^1,33^, we first aimed to investigate pneumonia phenotype patterns when discriminating CID against controls, both with pneumonia (Cohort 1). Among the top phenotypes, we identified pneumococcal and the broader bacterial pneumonia subtypes (Fig. 3a). In contrast to the general pneumonia phenotype, the above pneumonia subtypes did not precede PI diagnosis (Fig. 5). This might reflect lack of pneumonia categorization early in the CID spectrum. In Cohorts 2-4, to investigate the full spectrum of PI case profiles, the pneumonia filter was gradually removed from controls and patients (Fig. 1). In Cohort 2, there were similar pneumonia findings to Cohort 1, with non-pneumonia diseases dominating in Cohorts 3-4.

This study’s findings are clinically relevant to the medical community. First, we evaluated different perspectives of CID and CVID, by developing an accurate method and a comprehensive evaluation procedure across all 4 (CID/CVID) cohorts. Second, we developed a deep learning-based method that can learn nonlinearities due to large heterogeneities present in the data. Following evaluation, it can be possibly applied to other heterogeneous diseases, including other PI disorders. Third, we evaluated our model on a large cohort of global US patients (N = 47,660 participants). Fourth, since our analytical approach is based on the conversion of ICD codes to explicit clinical phenotypes and the statistical/ temporal analysis of phenotype combinations, it potentially has broad applicability for systematizing the identification of patients at risk for other underdiagnosed heterogeneous diseases and PI disorders.

Several limitations should be considered when interpreting our findings. The main limitation is reliance on ICD codes (medical claims) from EHR hence, cases and controls could sometimes be miscoded. However, model-derived PI identification was consistent across cohorts and the combinations revealed consisted of antecedent phenotypes that are widely reported in the literature^1–3,10–14^. Clinical history might be misinformed because of differences across regional, institutional, or individual ICD coding processes. The Optum data used for this study are EHR from numerous hospitals across US. Hence, any data content differences between hospitals, reflect nationally representative ICD coding processes. Converting into phenotypes may have minimized any biases from miscoded disease subtypes. Although we aimed to investigate diverse PI profiles across cohorts, we consistently identified congruent phenotypes between Cohorts 1-3 (CID) and Cohort 4 (CVID), which reflects the identification of consistent patterns in the presence of PI. Our dataset did not involve laboratory, genetic and imaging data, which could further enhance the diagnostic performance and clinical information. Our goal is to validate our method on external clinical medical claims data thus, investigating multi-modal data analyses is our future endeavor. Validation on clinical medical claims will also be important to evaluate the generalizability of our model to diverse external real-world data. The TabMLPNet with wide and deep components showed higher diagnostic performance against its deep-only and wide-only counterparts. Nonetheless, this difference was not significant for Cohorts 2 and 4, when compared against the TabMLPNet deep-only model. We will therefore continue evaluating both model variants in our future work. For CID, we focused on including all D81 ICD codes classified as “CID”, reported in the https://www.icd10data.com/ and https://icd10cmtool.cdc.gov/ websites (by excluding all SCID). In our future work, we will investigate additional PI subtypes (next to D81) that have been classified as “affecting cellular and humoral immunity” by the most recent International Union of Immunological Societies 2022 update on genotypic^34^ and phenotypic^35^ classification, such as from D80, D82 and D84 codes. ICD codes were more frequent for “Other CID and CVID” as well as “CID and CVID unspecified” (Supplementary Table 2) in our data, mainly because full PI characterization is commonly low in clinical settings due to imprecise ICD coding processes and a lack of referral to clinical immunologists^10^. It is possible that we already include other CID (next to D81) currently existing in the Optum data, under the general “Other CID” as well as “CID unspecified” codes; see co-occurring D80 and D84 confounding variables in the Supplementary Table 2. On that note, although we removed these as confounding variables from patient clinical history to avoid model bias, these patients were included in our analysis, as CID patients. Our data analysis has been performed on adult PI cases. One of our future directions is the concurrent analysis of both adult and pediatric data. Finally, our model should not be considered as a definitive method for the diagnosis of CID or CVID. Instead, it could be used as a starting point for potentially identifying adult patients at risk which can lead to an early referral to clinical immunologists and in turn access to appropriate blood, immunologic, genetic, imaging and other complementary medical assessments, to fully characterize and design treatments for CID/ CVID.

In conclusion, the proposed predictive scheme achieved accurate performance for the identification of CID and CVID based on a large-scale analysis performed on US representative medical claims. Our methodology can potentially lead to new clinical insights and expanded guidelines for the detection of phenotype combinations, increase clinical awareness and be used to improve identification of adults at risk as well as clinical outcomes on population level.

### Supplementary information

Supplementary_Information

Description of Additional Supplementary Files

Supplementary Data 1

Supplementary Data 2

Supplementary Data 3

Supplementary Data 4

Supplementary Data 5

Supplementary Data 6

Reporting summary

## Data Availability

Additional numerical values underlying Figs. 3 and 4 are presented in Supplementary Data 5. The datasets used for this study could not be made publicly available due to a data use commercial agreement between Pfizer and Optum. However, the authors encourage collaborations and would like to declare that the data can be made available to qualified investigators upon request with evidence of institutional review board approval.

## Code availability

We make available 5 main code pieces, which refer to the main data transformation and pre-processing steps up to machine learning model fitting (Supplementary Data 4): 1. Code used to convert ICD9 to ICD10 (SQL), 2. Convert ICD to the corresponding disease description (SQL), 3. Convert ICD to phenotypes (SQL), 4. Pivot table with features: ICD disease descriptions (Python) and 5. All machine learning models together with wide and deep, deep only and wide only experimental procedure (Python). We also give access to the 2018 GEMS mapping (Supplementary Data 1), to the processed Long, Short, Major and Sub Chapter descriptions of ICD codes (Supplementary Data 2) and to the phenotype mapping file that we created and used (Supplementary Data 3). The first data extraction step occurs directly inside Dataiku, by extracting diagnosis and patient data tables, as described in our Methods. Other code parts referring to data sanity checks is not publicly available for proprietary data reasons but can be made available to qualified investigators upon request.
