# Supplementary material for: Large-scale deep learning analysis to identify adult patients at risk for combined and common variable immunodeficiencies
Source: Commun Med (Lond). 2023 Dec 20;3:189. doi: 10.1038/s43856-023-00412-8 (PMC10733406; doi:10.1038/s43856-023-00412-8)
Supplement: Supplementary file 1 — Supplementary_Information [file 43856_2023_412_MOESM1_ESM.docx]

**Supplementary Information**

**Title: Large-scale deep learning analysis to identify adult patients at risk for combined and common variable immunodeficiencies**

**Authors:** Giorgos Papanastasiou,^1^ Guang Yang,^2,3,4^ Dimitris I. Fotiadis,^5,6^ Nikolaos Dikaios,^7^ Chengjia Wang,^8,9^ Ahsan Huda,^1^ Luba Sobolevsky,^10^ Jason Raasch,^11^ Elena Perez,^12^ Gurinder Sidhu,^1^ Donna Palumbo^1^

**Affiliations**

^1^ Pfizer Inc., New York, NY, USA

^2^ National Heart and Lung Institute, Imperial College London, London, UK

^3^ Cardiovascular Research Centre, Royal Brompton Hospital, London, UK

^4^ School of Biomedical Engineering & Imaging Sciences, King's College London, London, UK

^5^ Department of Biomedical Research, Institute of Molecular Biology and Biotechnology, FORTH, Ioannina, Greece

^6^ Unit of Medical Technology and Intelligent Information Systems, University of Ioannina, Ioannina, Greece

^7^ Mathematics Research Center, Academy of Athens, Athens, Greece

^8^ School of Mathematical and Computer Sciences, Heriot Watt, Edinburgh, UK

^9^ Edinburgh Centre for Robotics, Edinburgh, UK

^10^ Immunoglobulin National Society, Woodland Hills, CA, USA

^11^ Midwest Immunology Clinic, Plymouth, MN, USA

^12^ Allergy Associates of the Palm Beaches, North Palm Beach, FL, USA

**Supplementary Notes**

**Clinical phenotype importance**

Several phenotypes were revealed in Cohorts 1-4 (Figure 3). In Cohort 1, genetic carrier/susceptibility to disease, pneumococcal pneumonia, short stature, valvular heart disease and alveolar/parietoalveolar pneumonopathy were the five strongest phenotypes. Other respiratory (bronchiectasis, asthma), blood (decreased white blood cell (WBC) count, gram-negative septicemia) and autoimmunity conditions (disorders of the immune mechanism) were emerged (Figure 3a).

In Cohort 2, autoimmune disease not-elsewhere-classified (NEC), valvular heart disease, chromosomal anomalies, myopathy and non-Hodgkin lymphoma were the five strongest phenotypes. Similar respiratory (plus bronchopneumonia/ lung abscess), blood (plus thrombocytopenia) and autoimmunity conditions to Cohort 1, were revealed (Figure 3b).

In Cohort 3, deficiencies of circulating enzymes, autoimmune disease NEC, bone marrow/stem cell transplant, genetic carrier/susceptibility to disease and disorders of purine/pyrimidine metabolism were the top five phenotypes. Besides similar respiratory, blood and autoimmunity conditions to Cohorts 1-2, chromosomal anomalies, celiac disease, and unspecified or cardiac congenital anomalies emerged.

In the largest Cohort 4, chromosomal anomalies, disorders of purine/pyrimidine metabolism, chronic lymphocytic leukemia, deficiencies of circulating enzymes and bronchiectasis were the strongest phenotypes. Various other genetic (cleft palate, cardiac shunt heart septal defect, sarcoidosis), blood (thrombocytopenia, disease of WBC), autoimmunity (thymus gland, hypoparathyroidism) and blood cancer (non-Hodgkin lymphoma, lymphoid histiocytic tissue cancer) diseases were revealed.

**Temporal distribution of the earliest phenotypes**

The earliest three phenotypes were (median month value reported from Figure 4); Cohort 1: asthma(34.1), hypoparathyroidism(32.7), bone marrow/stem cell transplant(24.6); Cohort 2: autoimmune disease NEC(36.5), asthma(33.4), hypoparathyroidism(32.7); Cohort 3: psoriatic arthropathy(32.4), autoimmune disease NEC(28.8), paraproteinemia(25.8); Cohort 4: chronic lymphocytic leukemia(29.3), autoimmune disease NEC(23.7), non-Hodgkin lymphoma(21.7).

**Supplementary Tables**

**Supplementary Table 1)** The 10 Jeffrey Modell Foundation (JMF) warning signs of primary immunodeficiency (adapted from^7^).

| **Warning signs in children** |
| --- |
| 1.≥4 new ear infections within 1 year |
| 2.≥2 serious sinus infections within 1 year |
| 3.≥2 months on antibiotics with little effect |
| 4.≥2 pneumonias within 1 year |
| 5. Failure of an infant to gain weight or grow normally |
| 6. Recurrent, deep skin or organ abscesses |
| 7. Persistent thrush in mouth or fungal infection on skin |
| 8. Need for intravenous antibiotics to clear infections |
| 9.≥2 deep-seated infections including septicemia |
| 10. A family history of PI |

| **Warning signs in adults** |
| --- |
| 1.≥2 new ear infections within 1 year |
| 2.≥2 new sinus infections within 1 year, in the absence of allergy |
| 3. 1 pneumonia per year for>1 year |
| 4. Chronic diarrhea with weight loss |
| 5. Recurrent viral infections (colds, herpes, warts, condyloma) |
| 6. Recurrent need for intravenous antibiotics to clear infections |
| 7. Recurrent, deep abscesses of the skin or internal organs |
| 8. Persistent thrush or fungal infection on skin or elsewhere |
| 9. Infection with normally harmless tuberculosis-like bacteria |
| 10. A family history of PI |

| **ICD-10** | **ICD-9** | **Description** |  |
| --- | --- | --- | --- |
| **Combined immunodeficiencies (D81 ICD-10 section)** | | | **Prevalence %**  **(C3)** |
| D81.5 | 277.2 | Purine nucleoside phosphorylase deficiency | 4.42 |
| D81.6 | 279.2 | Major histocompatibility complex class I deficiency | 4.88 |
| D81.7 | 279.2 | Major histocompatibility complex class II deficiency | 1.46 |
| D81.810 | 277.6 | Biotinidase deficiency | 1.94 |
| D81.818 | 266.2 | Other biotin-dependent carboxylase deficiency | 2.81 |
| D81.819 | 266.2 | Biotin-dependent carboxylase deficiency, unspecified | 2.89 |
| D81.89 | 279.2 | Other combined immunodeficiencies | 11.61 |
| D81.9 | 279.2 | Combined immunodeficiency, unspecified | 69.99 |
| **D80 and D84 ICD-10 codes identified in the clinical history co-occurring with either D81.89 or D81.9** | | | **Prevalence %**  **(C3)** |
| D80.1 | 279.00 | Nonfamilial hypogammaglobulinemia | 2.58 |
| D80.2 | 279.01 | Selective deficiency of immunoglobulin A [IgA] | 1.23 |
| D80.3 | 279.03 | Selective deficiency of immunoglobulin G [IgG] | 2.98 |
| D80.4 | 279.02 | Selective deficiency of immunoglobulin M [IgM] | 1.31 |
| D80.6 | 279.09 | Antibody deficiency with near-normal immunoglobulins or hyperimmunoglobulinemia | 2.09 |
| D80.8 | 279.19 | Other immunodeficiencies with predominantly antibody defects | 4.61 |
| D80.9 | 279.09 | Immunodeficiency with predominantly antibody defects | 4.91 |
| D80.9 | 279.09 | Immunodeficiency with predominantly antibody defects unspecified | 2.93 |
| D84.8 | 279.10 | Other specified immunodeficiencies | 4.16 |
| D84.89 | 279.19 | Other immunodeficiencies | 6.60 |
| D84.9 | 279.3 | Immunodeficiency unspecified | 6.68 |
| **Common variable immunodeficiencies (D83 ICD-10 section)** | | | **Prevalence %**  **(C4)** |
| D83.0 | 279.06 | CVID with predominant abnormalities of B-cell numbers and function | 8.18 |
| D83.1 | 279.10 | CVID with predominant immunoregulatory T-cell disorders | 8.96 |
| D83.2 | 279.06 | CVID with autoantibodies to B- or T-cells | 2.05 |
| D83.8 | 279.06 | Other CVID | 10.84 |
| D83.9 | 279.06 | CVID, unspecified | 69.97 |

**Supplementary Table 2)** ICD diagnosis codes relevant to combined immunodeficiencies (CID) and common variable immunodeficiencies (CVID) that were present in our Optum® data^13^, across Cohorts 1-4 are shown. ICD-10 to ICD-9 conversions were based on 2018 GEMS. The right column presents the prevalence of each CID and CVID subtype in the largest Cohorts 3 (C3) and 4 (C4). D80 and D84 codes identified in the clinical history co-occurring with either D81.89 or D81.9 are also presented for C3 (these were removed as confounding variables to avoid biasing machine learning models). In C4, the percentage represents the prevalence by considering only the CVID patients. Note that some ICD codes for CID were not present at the time of extracting data, due to no prevalence. Patients across all ICD codes were identified for CVID, as CVID are more frequent compared to CID. Further information about CID and CVID codes were initially found in  [icd10data D81](https://www.icd10data.com/ICD10CM/Codes/D50-D89/D80-D89/D81-) and  [icd10data D83](https://www.icd10data.com/‌ICD10CM/Codes/D50-D89/D80-D89/D83-/D83), and confirmed by searching for relevant D81 and D83 ICD codes in the <https://icd10cmtool.cdc.gov/>.

**Supplementary Table 3) Diagnostic performance metrics in the testing set from separate comparison experiments between TabMLPNet with wide and deep against TabMLPNet with deep only and wide only, across Cohorts 1-4.** Note that a different testing set was used here against the experiments presented in Table 2. The ROC AUC for TabMLPNet wide and deep were significantly higher compared to TabMLPNet wide only across all cohorts (P values are indicated with * and were 0.005, 0.006, 0.01 and 0.05, respectively). The ROC AUC for TabMLPNet wide and deep were significantly higher compared to TabMLPNet deep only, in Cohorts 1 and 3 (P values are indicated with † and were 0.02 and 0.01, respectively). No other significant differences were observed between ROC curves.

| **Patient Cohorts** | | | | | |
| --- | --- | --- | --- | --- | --- |
| **Metric** | | **Cohort 1** | **Cohort 2** | **Cohort 3** | **Cohort 4** |
| Sensitivity | **TabMLPNet wd** | **0.87** | **0.88** | **0.81** | **0.85** |
|  | TabMLPNet d | 0.82 | 0.86 | 0.76 | 0.82 |
|  | TabMLPNet w | 0.82 | 0.82 | 0.79 | 0.81 |
| Specificity | **TabMLPNet wd** | **0.86** | **0.86** | **0.81** | **0.85** |
|  | TabMLPNet d | 0.83 | 0.84 | 0.75 | 0.82 |
|  | TabMLPNet w | 0.79 | 0.77 | 0.74 | 0.75 |
| PPV | **TabMLPNet wd** | **0.87** | **0.88** | **0.80** | **0.85** |
|  | TabMLPNet d | 0.82 | 0.86 | 0.76 | 0.82 |
|  | TabMLPNet w | 0.80 | 0.79 | 0.78 | 0.83 |
| NPV | **TabMLPNet wd** | **0.87** | **0.90** | **0.80** | **0.85** |
|  | TabMLPNet d | 0.82 | 0.88 | 0.76 | 0.82 |
|  | TabMLPNet w | 0.79 | 0.78 | 0.76 | 0.78 |
| Accuracy | **TabMLPNet wd** | **0.87** | **0.87** | **0.80** | **0.85** |
|  | TabMLPNet d | 0.82 | 0.85 | 0.76 | 0.82 |
|  | TabMLPNet w | 0.80 | 0.80 | 0.77 | 0.80 |
| ROC AUC | **TabMLPNet wd** | **0.93*** † | **0.94*** | **0.87*** † | **0.90*** |
|  | TabMLPNet d | 0.89 | 0.93 | 0.82 | 0.89 |
|  | TabMLPNet w | 0.86 | 0.85 | 0.83 | 0.85 |

| **Phenotype**  **Combination** | **Odds Ratio**  **(95% CI)** | **P values** | **Number of**  **phenotypes** |
| --- | --- | --- | --- |
| **Cohort 1** | | | |
| Genetic carrier/ susceptibility to disease;** Asthma;** Lack of normal PD UNS.** | 4.84 (1.72-7.96) | 0.0015 | 3 / 3 / 3 |
| Asthma;** Pneumonia;** Genetic carrier/ susceptibility to disease**. | 5.85 (3.43-8.27) | 0.0008 | 3 / 3 / 3 |
| Pneumococcal pneumonia; Disorders involving the IM;** Asthma.** | 5.98 (4.67-7.29) | 0.0008 | 3 / 2 / 2 |
| Other alveolar and parietoalveolar pneumonopathy; Bacterial pneumonia; Asthma.** | 6.13 (4.39-7.87) | 0.0005 | 3 / 1 / 1 |
| Valvular heart disease/ heart chambers;** ENC for LT use of MED;* Asthma.** | 6.46 (5.68-7.24) | 0.0004 | 3 / 3 / 2 |
| Lack of normal PD UNS;** Valvular heart disease/ heart chambers;** Pneumonia.** | 5.78 (4.02-7.54) | 0.0008 | 3 / 3 / 3 |
| Decreased WBC count;** Asthma.** | 5.41 (3.72-7.10) | 0.0009 | 2 / 2 / 2 |
| Decreased WBC count;** Bacterial pneumonia; ENC for long term use of MED;* Asthma.** | 6.97 (4.89-9.05) | 0.0001 | 4 / 3 / 2 |
| Bronchiectasis;** Decreased WBC count;** ENC for long term use of MED.* | 5.75 (3.45-8.05) | 0.0008 | 3 / 3 / 2 |
| Disorders involving the IM;** Decreased WBC count;** Asthma.** | 6.53 (2.22-8.75) | 0.0003 | 3 / 3 / 3 |
| Decreased WBC count;** Disorders involving the IM;**  Bacterial pneumonia; ENC for LT use of MED;* Asthma.** | 4.98 (3.23-6.73) | 0.0013 | 5 / 4 / 3 |
| **Cohort 2** | | | |
| Autoimmune disease NEC;** Pneumonia;** Fever of unknown origin.** | 5.79 (3.34-8.24) | 0.0008 | 3 / 3 / 3 |
| Valvular heart disease/ heart chambers;** Asthma;** Fever of unknown origin.** | 5.30 (2.94-7.66) | 0.0009 | 3 / 3 / 3 |
| Chromosomal anomalies;** Pneumonia;** Valvular heart disease/ heart chambers.** | 5.22 (3.88-6.56) | 0.0009 | 3 / 3 / 3 |
| Myopathy;** Pneumonia;** Asthma.** | 5.32 (3.26-7.38) | 0.0009 | 3 / 3 / 3 |
| Non-Hodgkin lymphoma;** Pneumonia;** Fever of unknown origin.** | 6.96 (3.76-10.20) | 0.0001 | 3 / 3 / 3 |
| Thrombocytopenia;* Non-Hodgkin lymphoma;** Pneumonia.** | 6.75 (3.49-10.01) | 0.0001 | 3 / 3 / 2 |
| Pneumococcal pneumonia; Non-Hodgkin lymphoma;** Fever of unknown origin.** | 5.88 (3.74-8.01) | 0.0008 | 3 / 2 / 2 |
| Disorders involving the immune mechanism;** Chromosomal anomalies;**  Fever of unknown origin.** | 5.25 (3.73-6.77) | 0.0009 | 3 / 3 / 3 |
| Asthma;** ENC for long term use of antibiotics; Fever of unknown origin.** | 6.33 (3.27-9.39) | 0.0002 | 3 / 2 / 2 |
| Bone marrow /stem cell transplant;** Decreased WBC count;** Pneumonia.** | 5.65 (3.47-7.83) | 0.0009 | 3 / 3 / 3 |
| Myeloproliferative disease;** Asthma;** Fever of unknown origin.** | 5.89 (3.23-8.54) | 0.0008 | 3 / 3 / 3 |

**Supplementary Table 4) Top 11 combinations of clinical phenotypes and their association with PI (Cohorts 1 and 2).** The table presents combinations which had at least one phenotype in addition to PI. Phenotypes were selected hierarchically (based on ORs; see Figures 3-6), introducing at least one new phenotype combination in each table row (per cohort) and by including the highest number of possible combinations with significant ORs>3.00. The X/ Y/ Z numbering indicates the total number of phenotypes in each combination and how many of these had a median of first diagnosis greater than 3 (indicated with *) and 6 months (**) before PI diagnosis, respectively. PD: Physiological development; UNS: Unspecified; ENC: Encounter; LT: Long-term; MED: Medications; WBC: White blood cell; IM: Immune mechanism; NEC: Not elsewhere classified. Note: valvular heart disease/ heart chambers involve all types of valve diseases and undefined cardiomyopathy.

| **Phenotype**  **Combination** | **Odds Ratio**  **(95% CI)** | **P values** | **Number of**  **phenotypes** |
| --- | --- | --- | --- |
| **Cohort 3** | | | |
| Other deficiencies of circulating enzymes; Disorders involving the IM.** | 4.86 (2.73-6.93) | 0.0013 | 2 / 1 / 1 |
| Autoimmune disease NEC;** Asthma.** | 4.76 (2.33-7.14) | 0.0014 | 2 / 2 / 2 |
| Bone marrow /stem cell transplant;** Disorders involving the IM;** Asthma.** | 6.38 (4.67-8.29) | 0.0003 | 3 / 3 / 3 |
| Pneumonia;** Bone marrow /stem cell transplant;** Disorders involving the IM.** | 6.35 (3.87-8.84) | 0.0003 | 3 / 3 / 3 |
| Bone marrow /stem cell transplant;** Chromosomal anomalies. | 5.46 (3.68-7.24) | 0.0009 | 2 / 1 / 1 |
| Genetic carrier /susceptibility to disease;* Asthma.** | 6.78 (5.02-8.54) | 0.0001 | 2 / 2 / 1 |
| Genetic carrier /susceptibility to disease;* Other disorders of purine and pyrimidine metabolism. | 5.81 (4.72-6.89) | 0.0008 | 2 / 1 / 0 |
| Celiac disease;** Asthma.** | 5.37 (2.89-7.85) | 0.0009 | 2 / 2 / 2 |
| Decreased WBC count;* Pneumonia;** Asthma.** | 5.75 (4.45-7.04) | 0.0008 | 3 / 3 / 2 |
| Decreased WBC count;* Cardiac congenital anomalies.* | 6.83 (4.22-9.44) | 0.0001 | 2 / 2 / 0 |
| Perforation of tympanic membrane;** Asthma.** | 5.58 (4.23-6.92) | 0.0009 | 2 / 2 / 2 |
| **Cohort 4** | | | |
| Chromosomal anomalies;* Cardiac congenital anomalies; Lack of normal PD UNS. | 6.09 (5.75-6.43) | 0.0005 | 3 / 1 / 0 |
| Disorders involving the IM;* Non-Hodgkin lymphoma;** Asthma.** | 6.07 (5.71-6.44) | 0.0005 | 3 / 3 / 2 |
| Bone marrow /stem cell transplant;** Disorders involving the IM.* | 5.86 (5.31-6.41) | 0.0008 | 2 / 2 / 1 |
| Autoimmune disease NEC;** Celiac disease.** | 5.75 (4.03-6.52) | 0.0008 | 2 / 2 / 2 |
| Bone marrow /stem cell transplant;** Bronchiectasis;* Non-Hodgkin lymphoma.** | 5.98 (3.75-8.21) | 0.0008 | 3 / 3 / 2 |
| Autoimmune disease NEC;** Asthma.** | 5.35 (3.51-7.19) | 0.0009 | 2 / 2 / 2 |
| Autoimmune disease NEC;** Celiac disease;** Disorders involving the IM.* | 4.56 (2.85-6.27) | 0.0012 | 3 / 3 / 2 |
| Psoriatic arthropathy;** Autoimmune disease NEC;** Asthma.** | 6.25 (4.73-7.77) | 0.0002 | 3 / 3 / 3 |
| Psoriatic arthropathy;** Disorders involving the IM;* Asthma.** | 5.33 (3.27-7.39) | 0.0009 | 3 / 3 / 2 |
| Non-Hodgkin lymphoma;** Psoriatic arthropathy;** Disorders involving the IM.* | 5.65 (4.47-6.83) | 0.0009 | 3 / 3 / 2 |
| Disorders involving the IM;* Non-Hodgkin lymphoma.** | 5.29 (4.23-6.34) | 0.0009 | 2 / 2 / 1 |

**Supplementary Table 5) Top 11 combinations of clinical phenotypes and their association with PI (Cohorts 3 and 4).** The table presents combinations which had at least one phenotype in addition to PI. Phenotypes were selected hierarchically (based on ORs; see Figures 3-6), introducing at least one new phenotype combination in each table row (per cohort) and by including the highest number of possible combinations with significant ORs>3.00. The X/ Y/ Z numbering indicates the total number of phenotypes in each combination and how many of these had a median of first diagnosis greater than 3 (indicated with *) and 6 months (**) before PI diagnosis, respectively. PD: Physiological development; UNS: Unspecified; ENC: Encounter; LT: Long-term; MED: Medications; WBC: White blood cell; IM: Immune mechanism; NEC: Not elsewhere classified. Note: valvular heart disease/ heart chambers involve all types of valve diseases and undefined cardiomyopathy.

**Supplementary Figures**


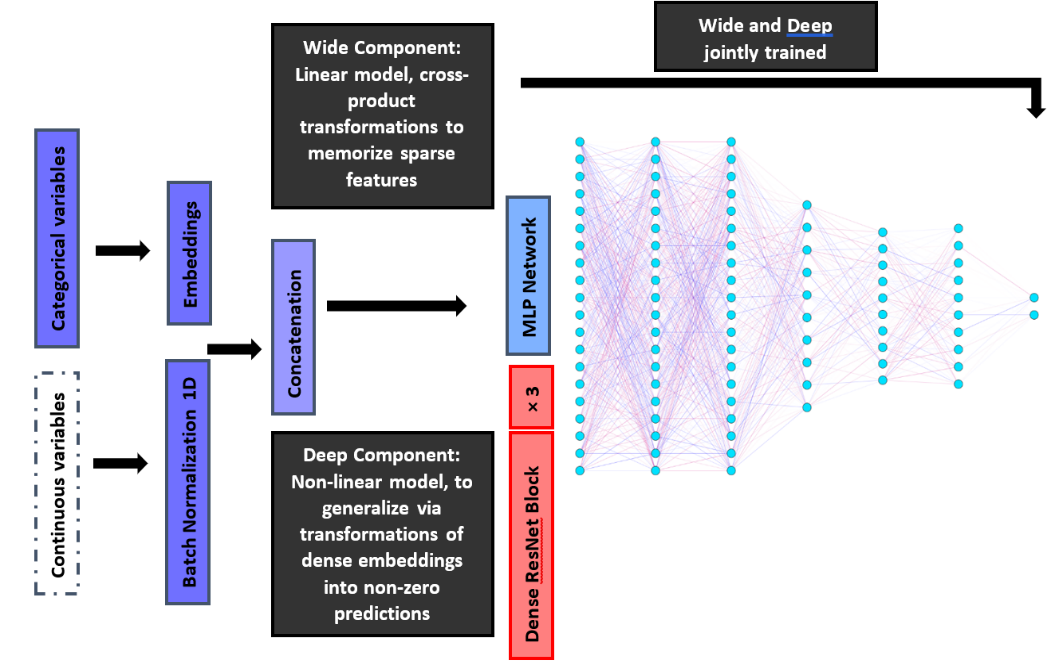


a)


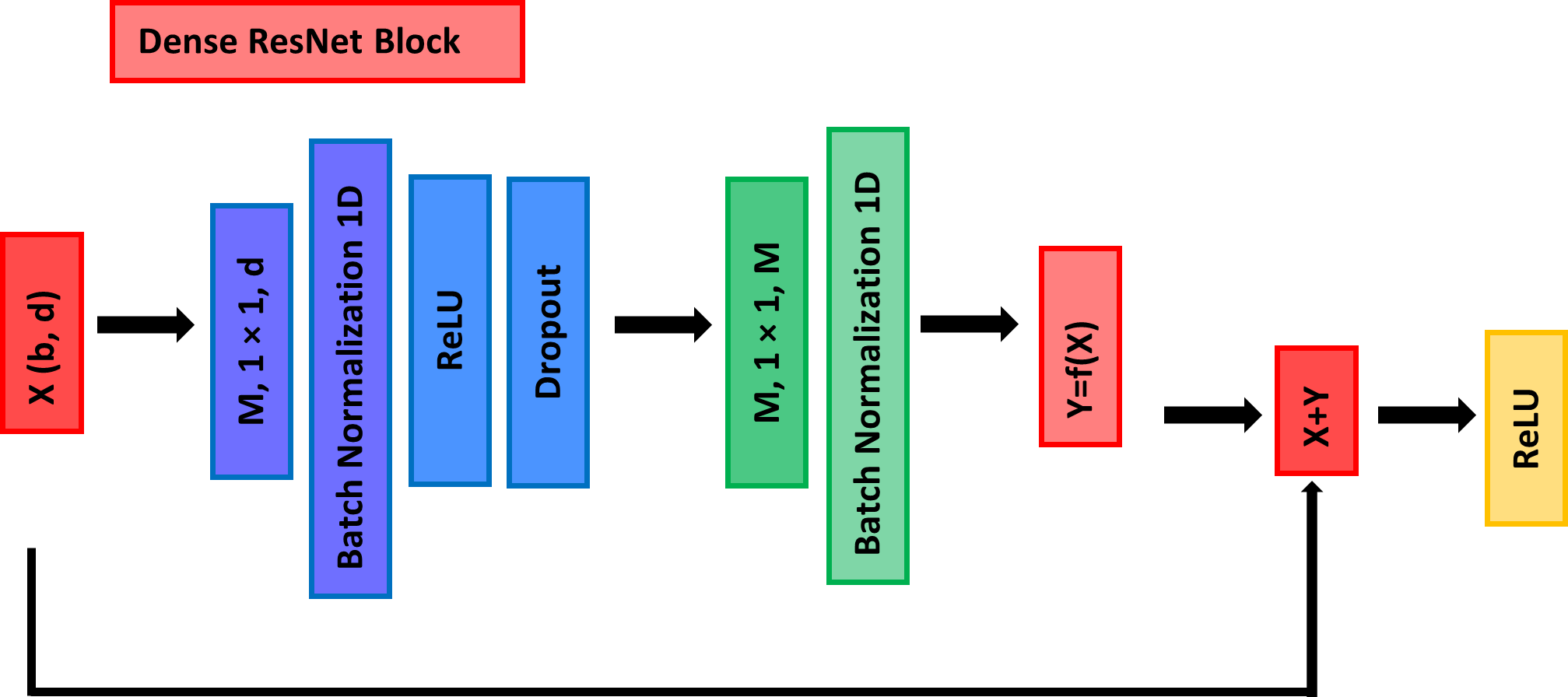


b)

**Supplementary Figure 1) TabMLPNet and TabResNet wide and deep learning model architectures.** Categorical variables were processed in our study by using binary values across diagnostic (ICD) features. The wide and deep components are jointly trained for both models^19,20^. The deep components are essentially feed-forward neural networks as shown in Figure 1a. The wide component is designed to memorize sparse features via cross-product transformations (see next Supplementary Figure 2)^19^. The TabMLPNet model is produced by eliminating the dense ResNet block from the TabResNet model (Figure 1b). Further architectural details (such as dimension and number of layers per model) are described in the Methods.


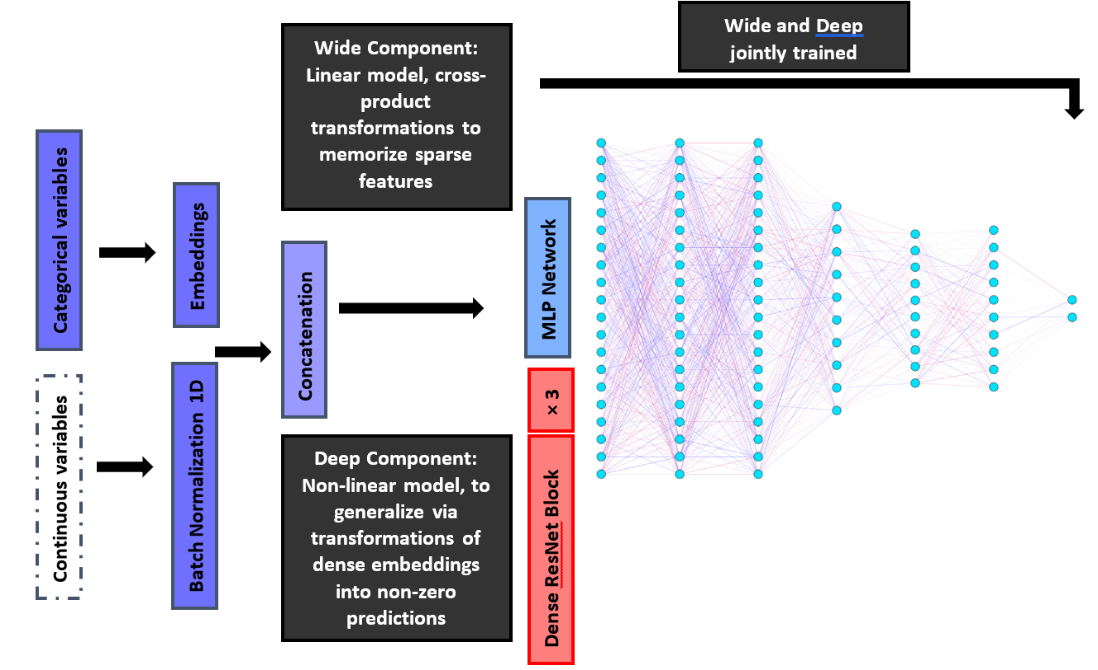

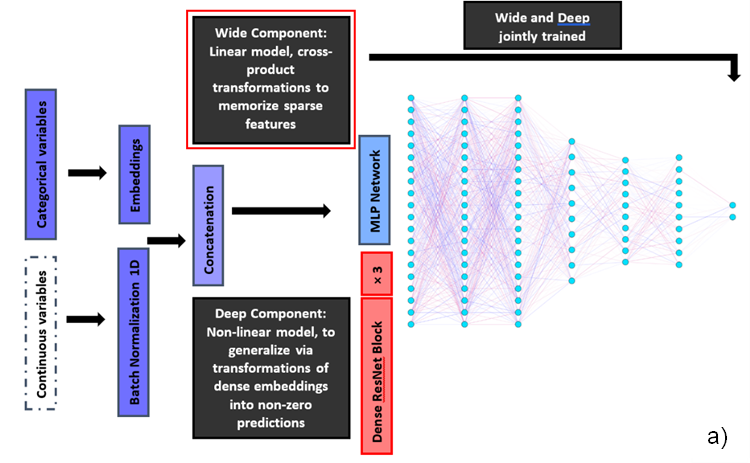

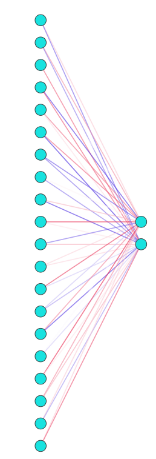


The wide component is a **generalized linear model** of the form^19^:

$y=w^{T}\cdot x+b$

where y is the prediction, x = [x_1_, x_2_,…,x_z_] is a vector of z features, w = [w_1_, w_2_,…,w_z_] are the estimated model weights and b is the bias.

The main learning process of the wide component is based on cross-product transformations, defined as follows:

$$\varphi_{k}\left( x \right)=\prod_{i=1}^{z} x_{i}^{c_{ki}} c_{ki}\in\left\{ 0, 1 \right\}$$

where c_ki_ is a Boolean variable which is 1 or 0 if the i-th feature is present or not respectively, in the k-th transformation φ_k_. For example, when categorical features are involved such as “pneumonia subtype X” and “autoimmune disease subtype Y”, a cross-product transformation is 1 if and only if the constituent features “pneumonia subtype X” and “autoimmune disease subtype Y” are both 1, and 0 otherwise.

**Supplementary Figure 2) The wide component in TabMLPNet and TabResNet** **models**.


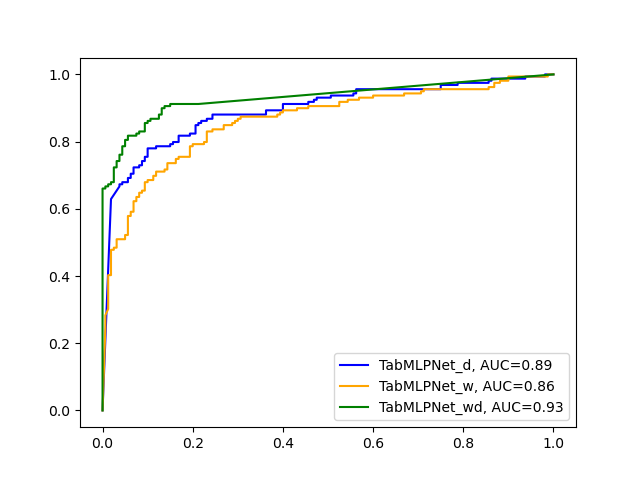

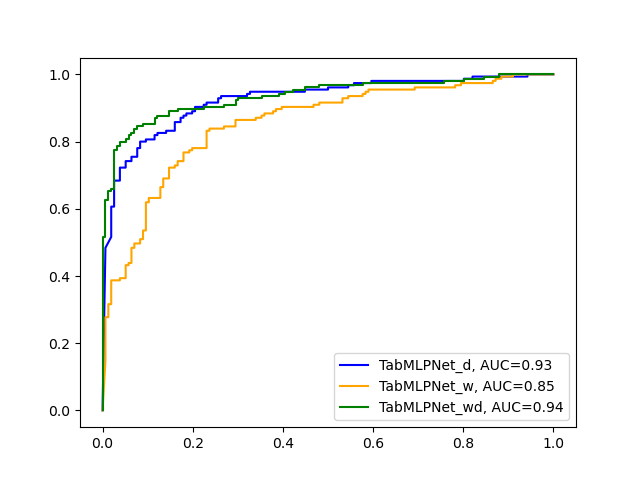

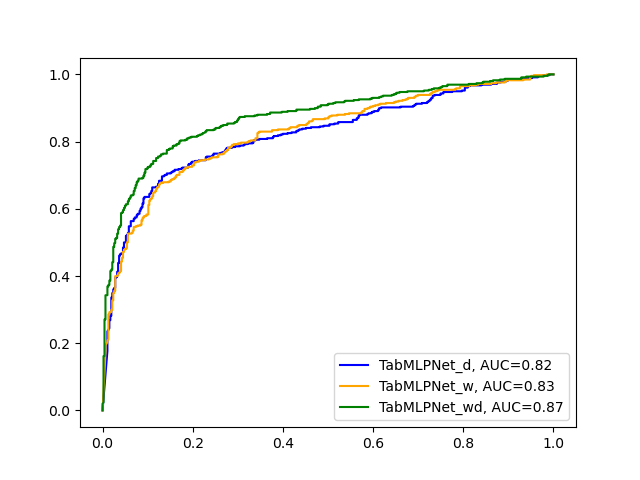

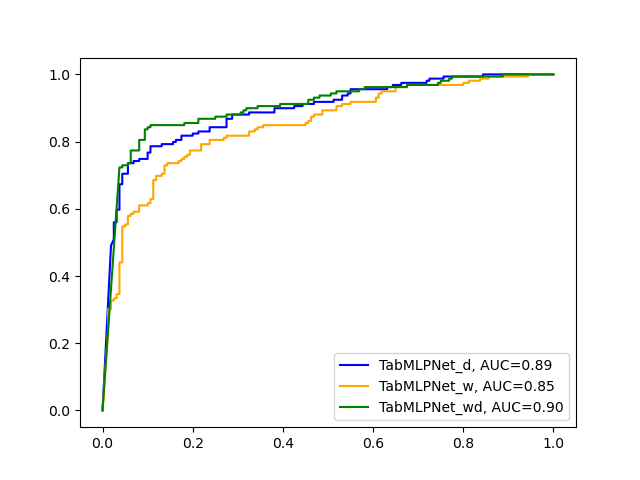


a)

b)

c)

d)

**1-Specificity**

**1-Specificity**

**1-Specificity**

**1-Specificity**

**Sensitivity**

**Sensitivity**

**Sensitivity**

**Cohort 1**

**Cohort 2**

**Cohort 3**

**Cohort 4**

**Sensitivity**

**Sensitivity**

**Supplementary Figure 3 a-d) Separate comparison experiments between TabMLPNet with wide and deep against TabMLPNet with deep only and wide only, across Cohorts 1-4, respectively.** TabMLPNet_d, TabMLPNet_w and TabMLPNet_wd denote deep only, wide only, and wide & deep TabMLPNet model variants, respectively.

**
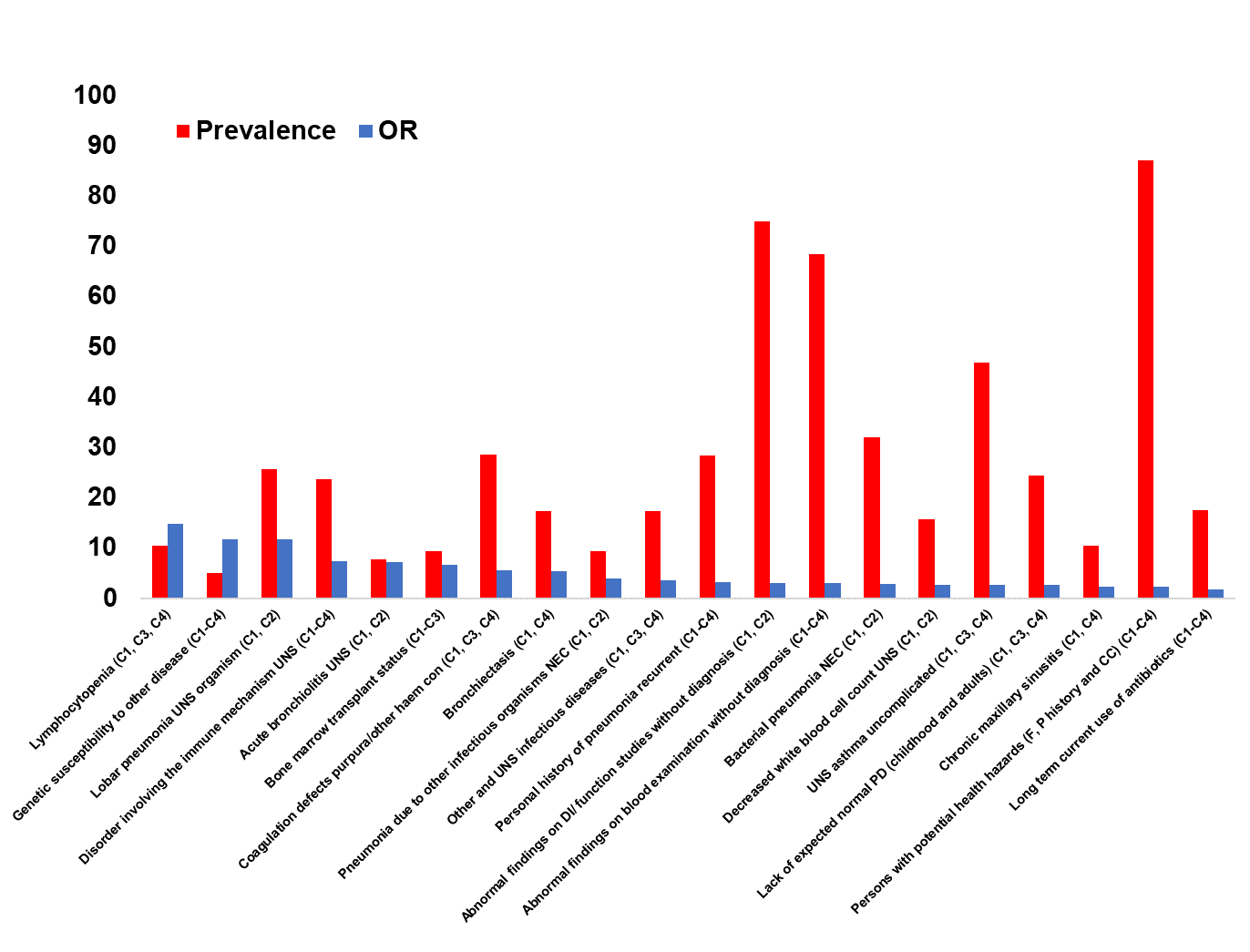
**

**Supplementary Figure 4) Odds ratio (OR, blue) and prevalence (%, red) for the top ICD codes (named based on their disease description) associated with CID in Cohort 1.** All clinical phenotypes significantly associated with the diagnosis of PI that had an OR > 1.5 were included in the illustrations. Univariate logistic regression was used to calculate the ORs. PMN: Polymorphonuclear neutrophils, F: Family, P: Personal, CC: Certain conditions, PD: Physiological development, UNS: Unspecified, DI: Diagnostic imaging, NEC: Not elsewhere classified, LH: Lymphoid hematopoietic, RC: Respiratory and cardiovascular, CID: Combined immunodeficiencies, CVID: Common variable immunodeficiencies, C1-C4: Declare congruent ICD codes in Cohorts 1-4.

**
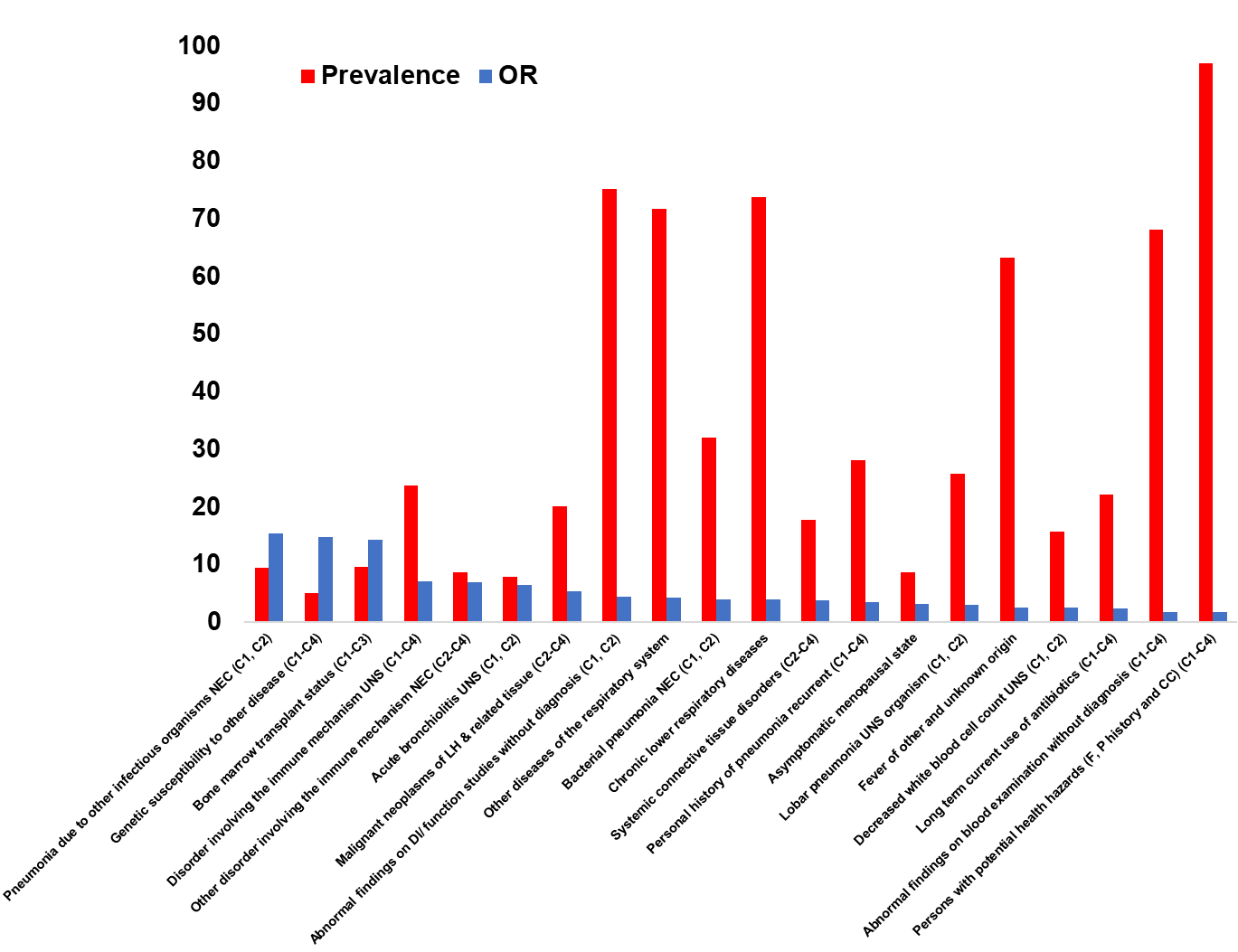
**

**Supplementary Figure 5) Odds ratio (OR, blue) and prevalence (%, red) for the top ICD codes (named based on their disease description) associated with CID in Cohort 2.** All clinical phenotypes significantly associated with the diagnosis of PI that had an OR > 1.5 were included in the illustrations. Univariate logistic regression was used to calculate the ORs. PMN: Polymorphonuclear neutrophils, F: Family, P: Personal, CC: Certain conditions, PD: Physiological development, UNS: Unspecified, DI: Diagnostic imaging, NEC: Not elsewhere classified, LH: Lymphoid hematopoietic, RC: Respiratory and cardiovascular, CID: Combined immunodeficiencies, CVID: Common variable immunodeficiencies, C1-C4: Declare congruent ICD codes in Cohorts 1-4.

**
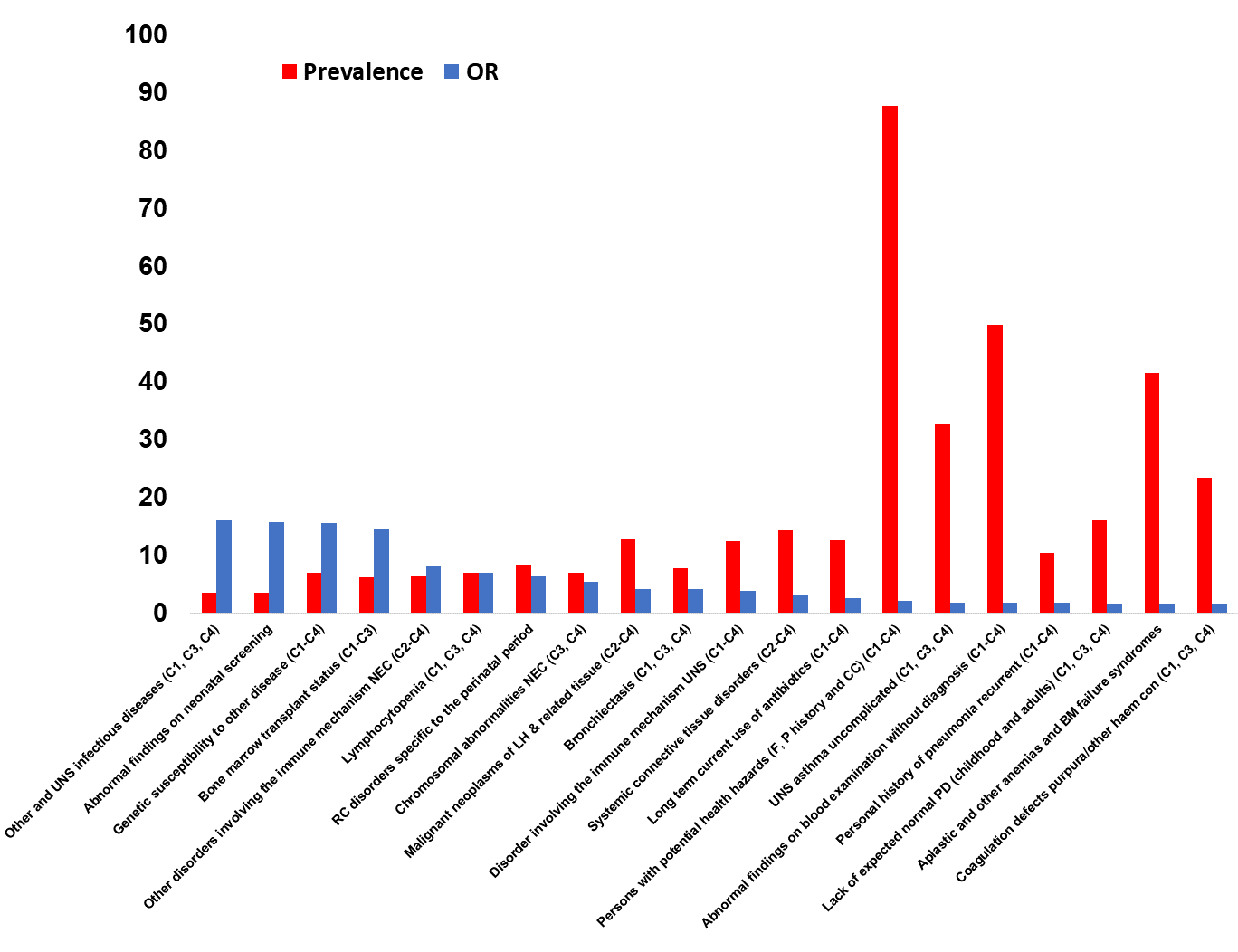
**

**Supplementary Figure 6) Odds ratio (OR, blue) and prevalence (%, red) for the top ICD codes (named based on their disease description) associated with CID in Cohort 3.** All clinical phenotypes significantly associated with the diagnosis of PI that had an OR > 1.5 were included in the illustrations. Univariate logistic regression was used to calculate the ORs. PMN: Polymorphonuclear neutrophils, F: Family, P: Personal, CC: Certain conditions, PD: Physiological development, UNS: Unspecified, DI: Diagnostic imaging, NEC: Not elsewhere classified, LH: Lymphoid hematopoietic, RC: Respiratory and cardiovascular, CID: Combined immunodeficiencies, CVID: Common variable immunodeficiencies, C1-C4: Declare congruent ICD codes in Cohorts 1-4.

**
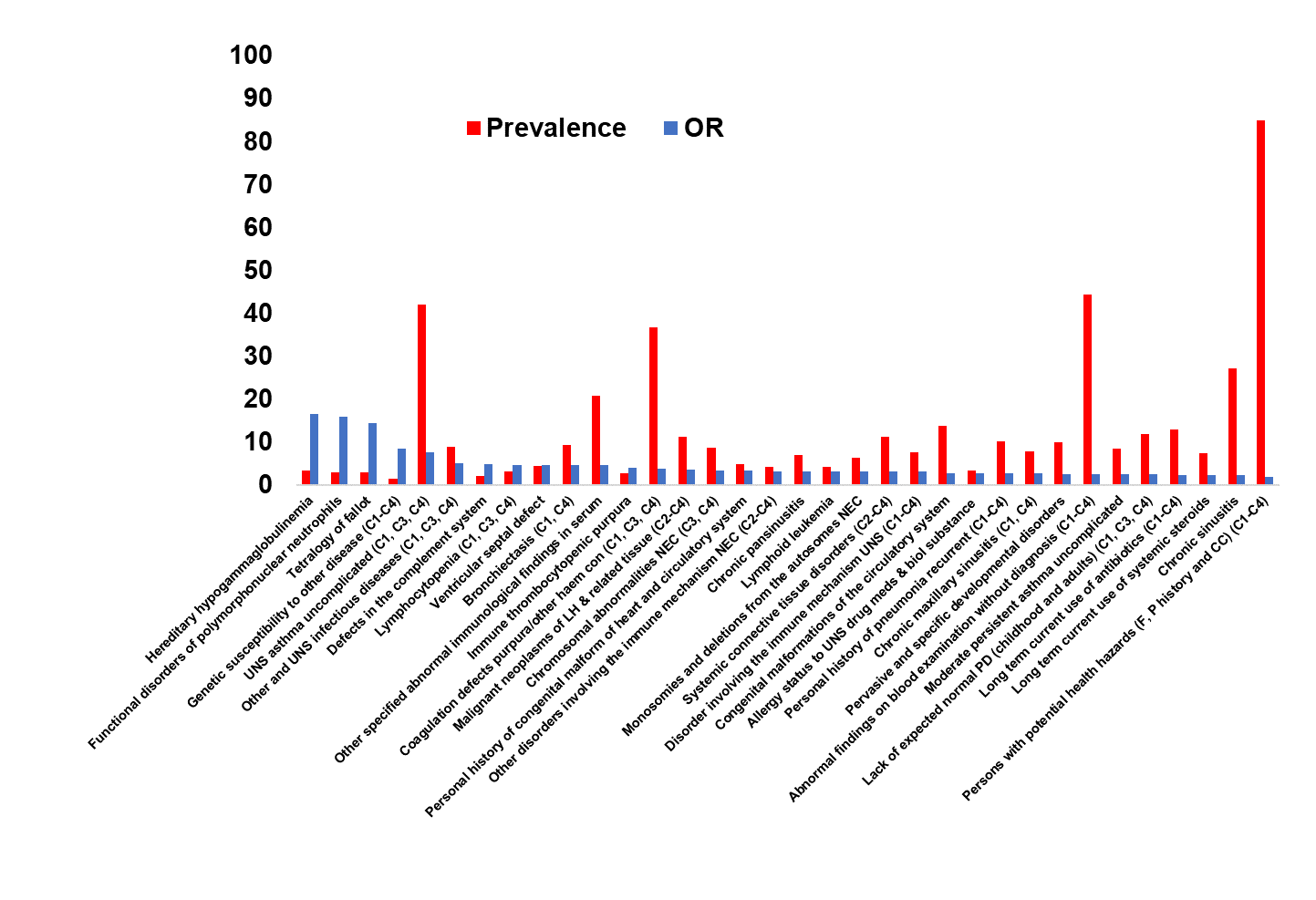
**

**Supplementary Figure 7) Odds ratio (OR, blue) and prevalence (%, red) for the top ICD codes (named based on their disease description) associated with CID and CVID in Cohort 4.** All clinical phenotypes significantly associated with the diagnosis of PI that had an OR > 1.5 were included in the illustrations. Univariate logistic regression was used to calculate the ORs. PMN: Polymorphonuclear neutrophils, F: Family, P: Personal, CC: Certain conditions, PD: Physiological development, UNS: Unspecified, DI: Diagnostic imaging, NEC: Not elsewhere classified, LH: Lymphoid hematopoietic, RC: Respiratory and cardiovascular, CID: Combined immunodeficiencies, CVID: Common variable immunodeficiencies, C1-C4: Declare congruent ICD codes in Cohorts 1-4.

**Years to Pneumonia diagnosis**

**Years to Pneumonia diagnosis**

**Proportion (%)**


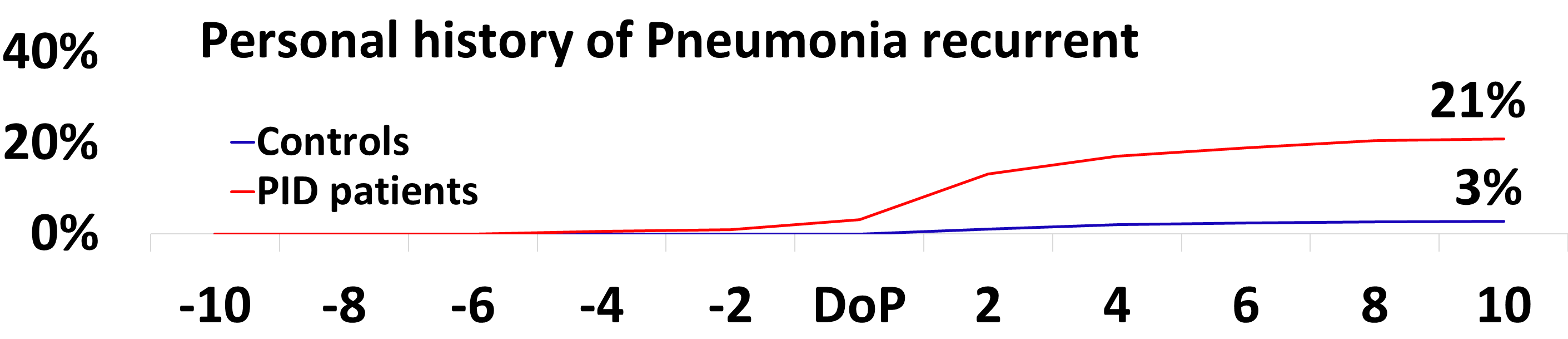

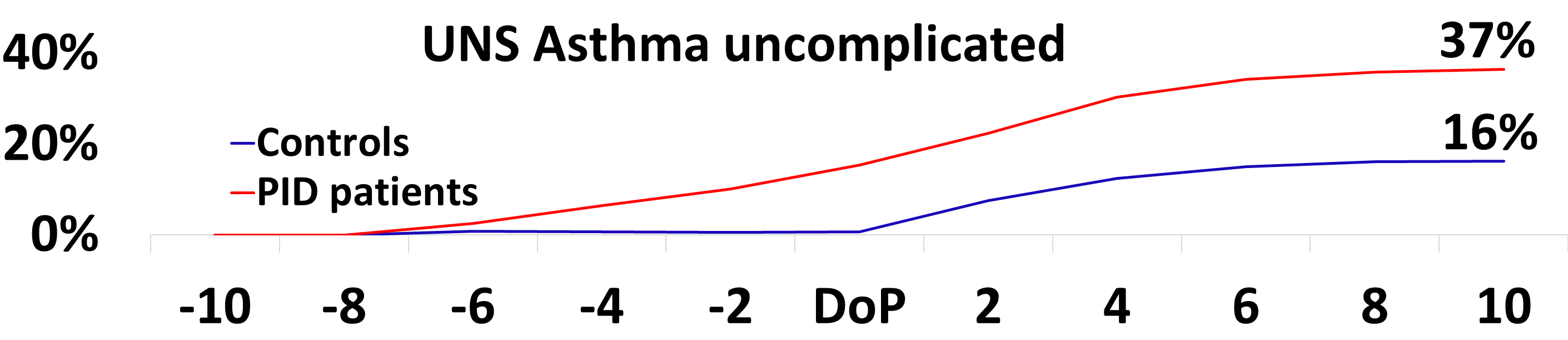


**a)**

**b)**


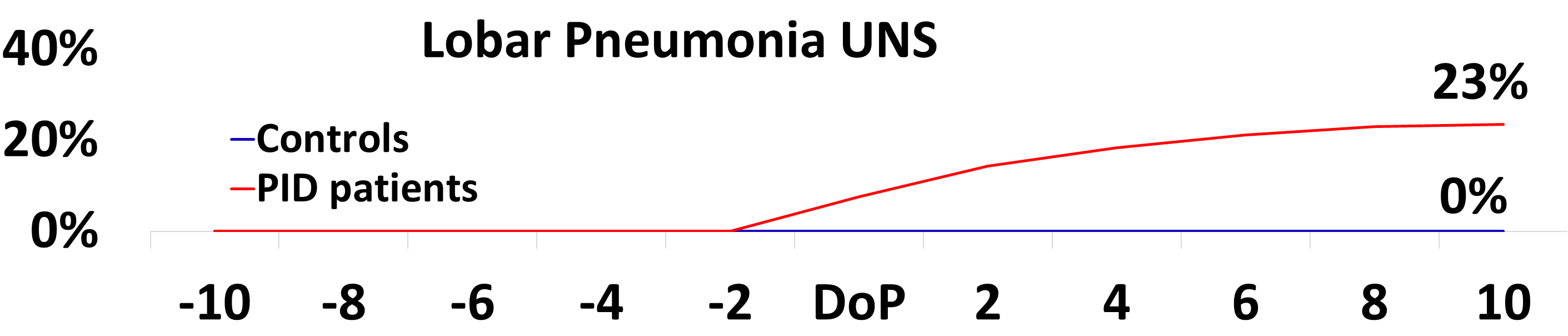


**Years to Pneumonia diagnosis**

**Years to Pneumonia diagnosis**

**Proportion (%)**


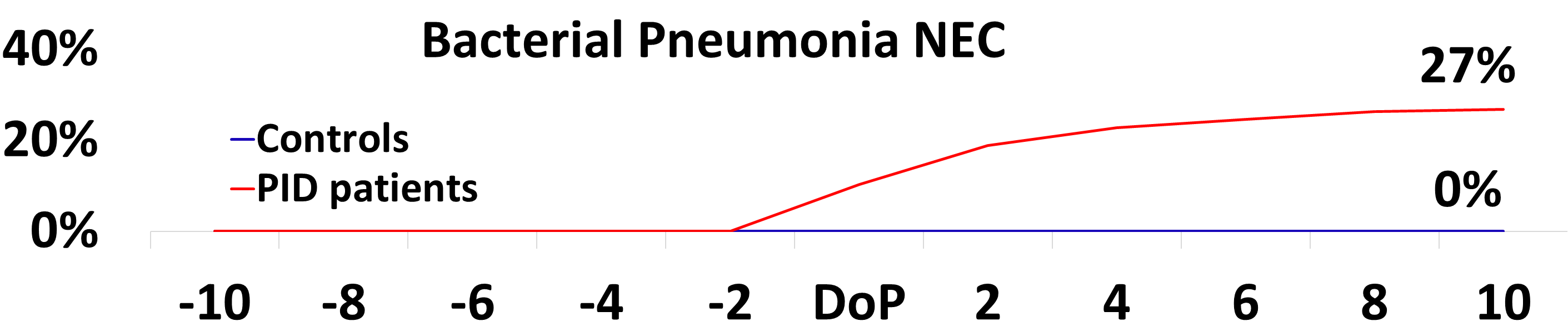


**c)**

**d)**


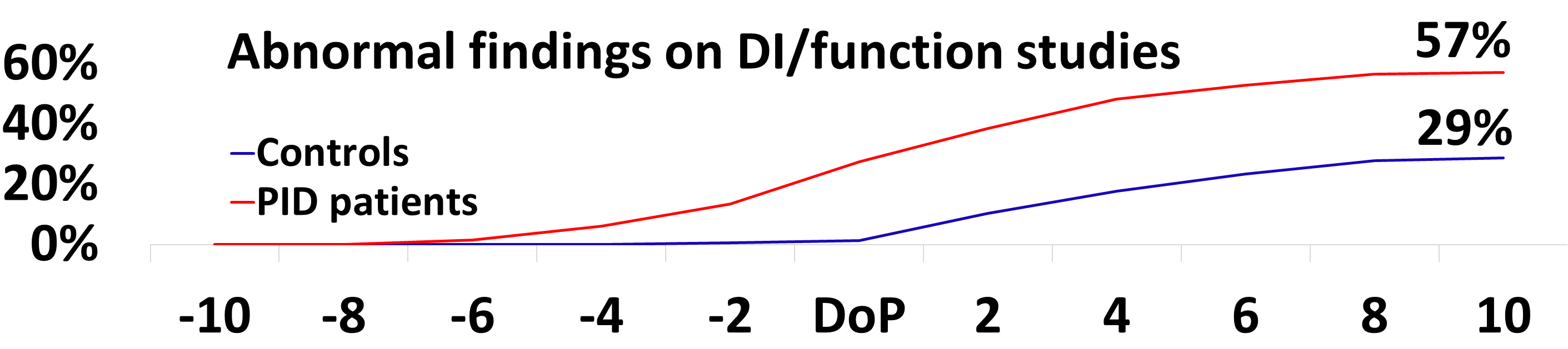


**Years to Pneumonia diagnosis**

**Proportion (%)**


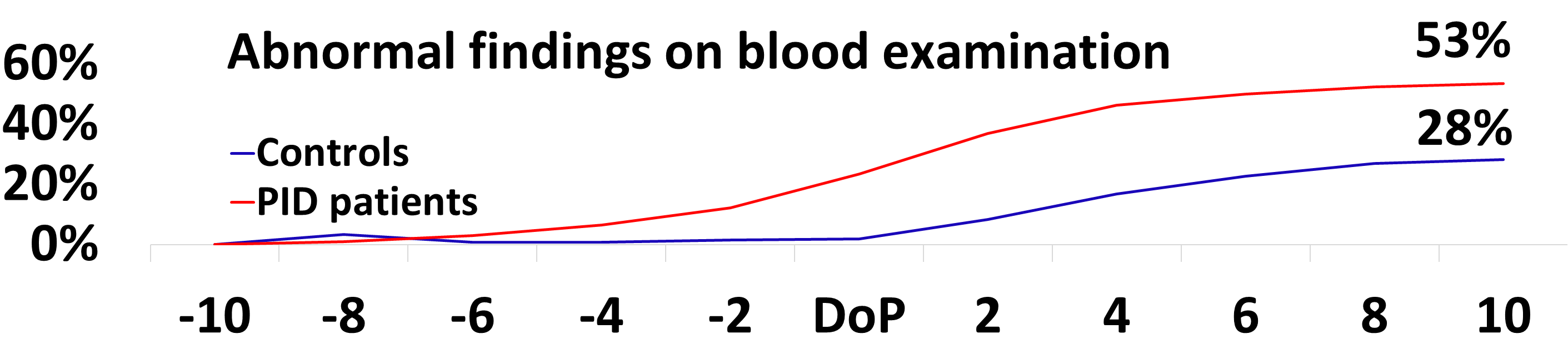


**Years to Pneumonia diagnosis**

**e)**

**f)**


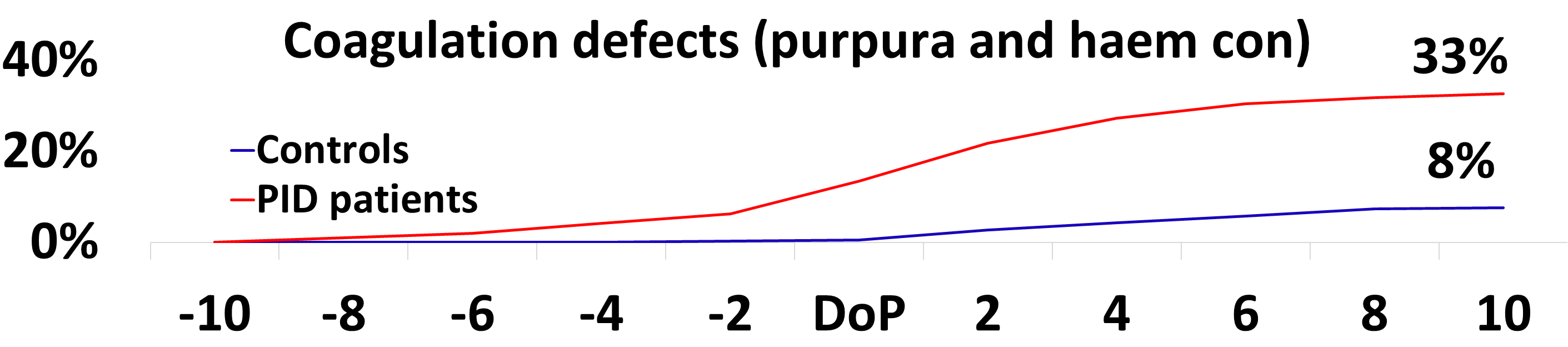


**Years to Pneumonia diagnosis**

**Proportion (%)**

**Years to Pneumonia diagnosis**


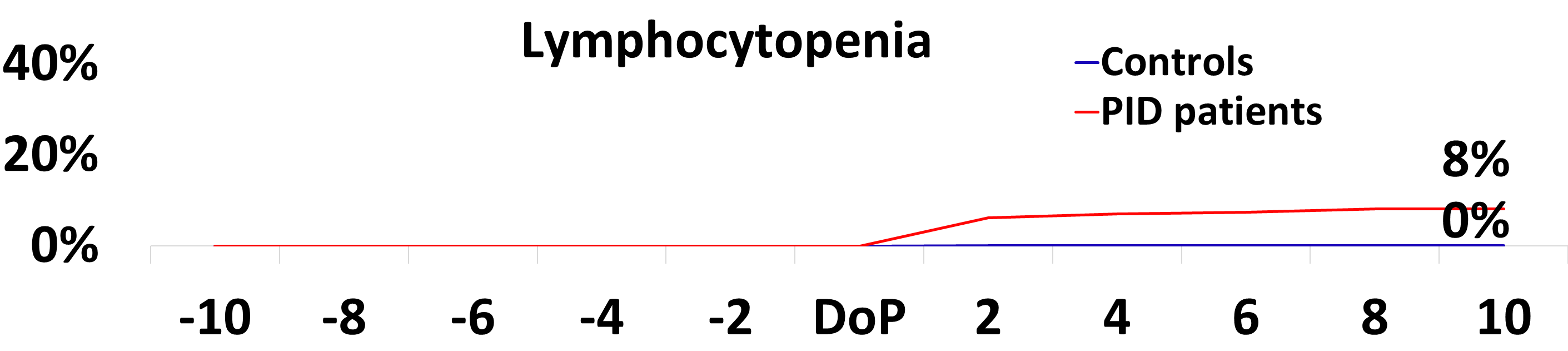


**g)**

**h)**

**Proportion (%)**

**Years to Pneumonia diagnosis**

**Years to Pneumonia diagnosis**


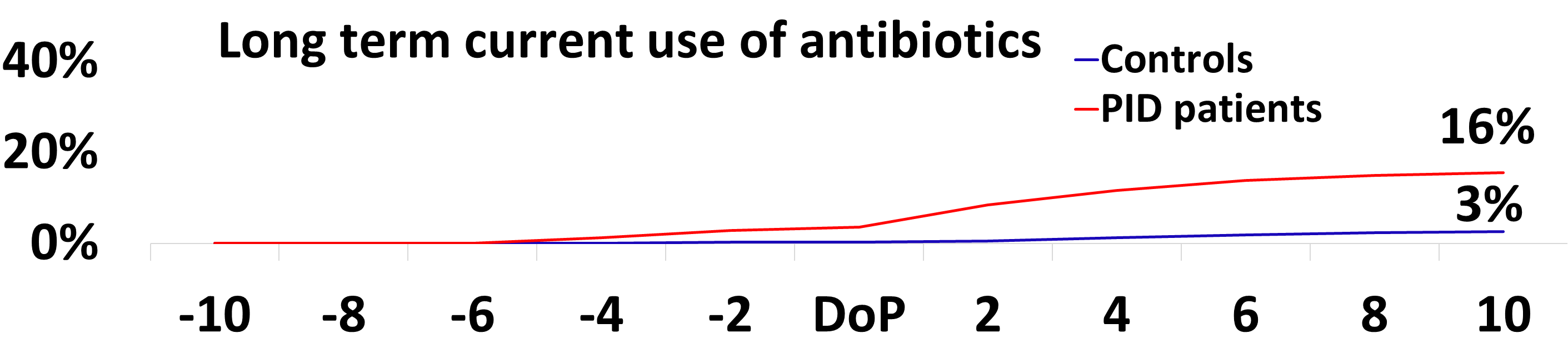

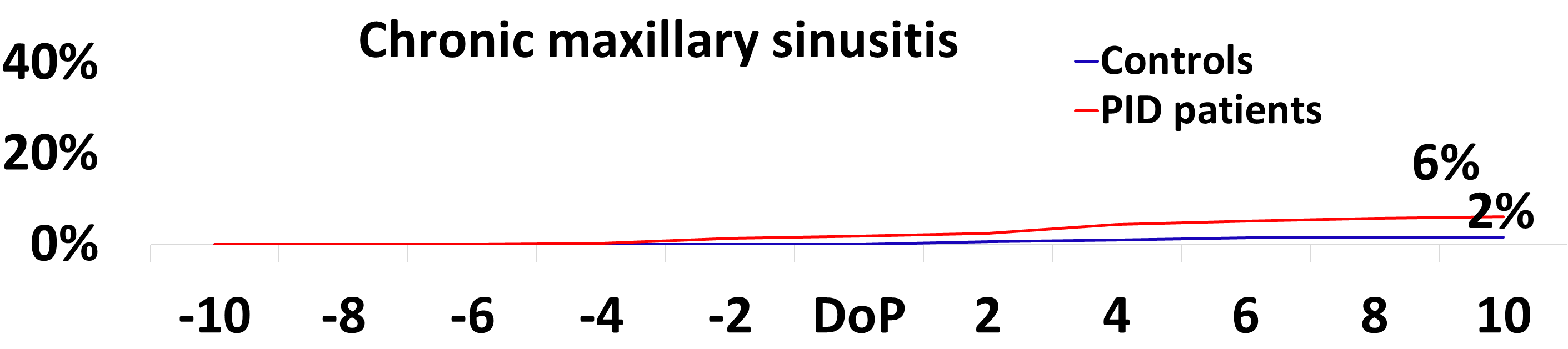


**i)**

**j)**

**Supplementary Figure 8) 20-year time frames of pulmonary (a-d) and non-pulmonary (e-j) ICD codes associated with PI (CID) patients with pneumonia against non-PI patients with pneumonia, prior to and after the diagnosis of pneumonia** (used as a common phenotype between PI cases and controls).

The illustrations depict the cumulative proportion of patients with each particular ICD code, which equals the sum of the proportions from each of the years preceding or following pneumonia diagnosis. CID: Combined immunodeficiency, DoP: Diagnosis of pneumonia, UNS: Unspecified, NEC: Not elsewhere classified, DI: Diagnostic imaging, haem con: Hemorrhagic conditions. Note that both e) and f) represent diagnostic procedures “without diagnosis”. The x axis shows years.

**Years to Pneumonia diagnosis**

**Years to Pneumonia diagnosis**

**Proportion (%)**


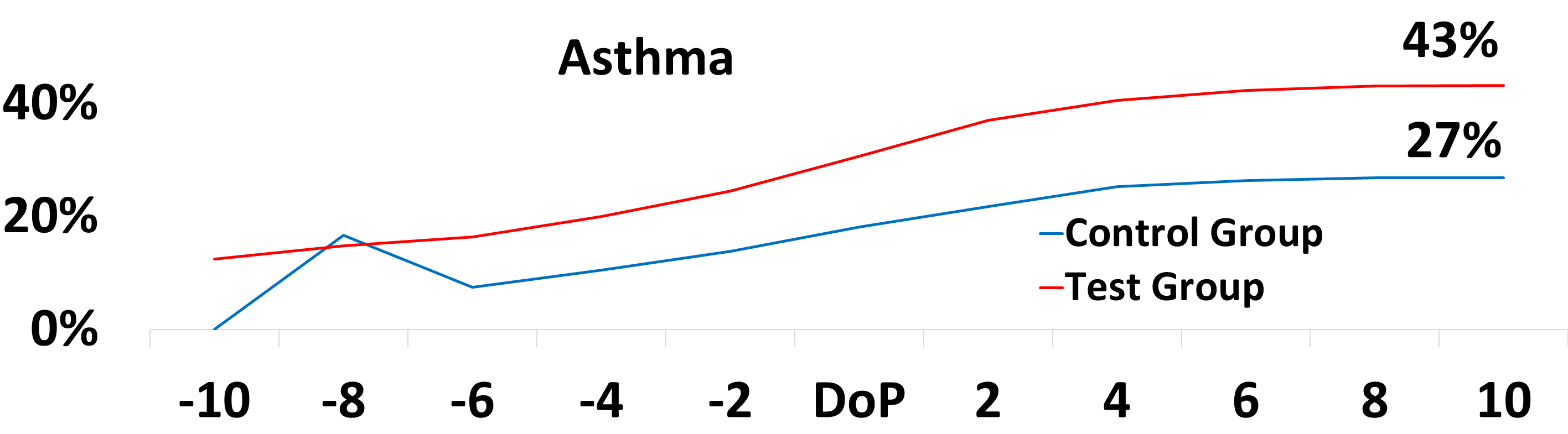

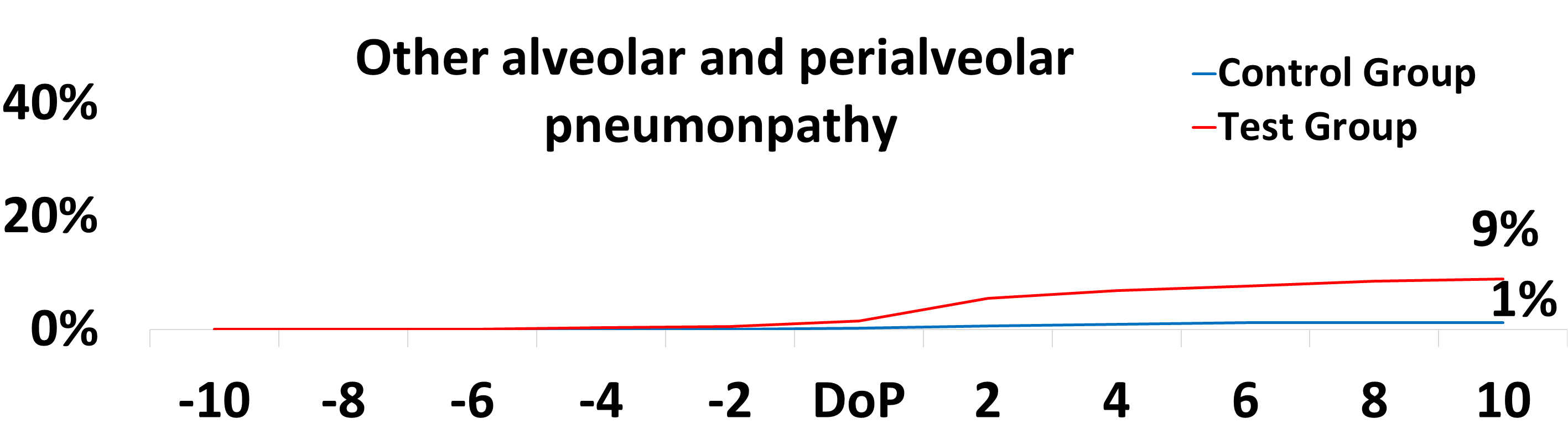


**d)**

**c)**

**Years to Pneumonia diagnosis**

**Years to Pneumonia diagnosis**

**Proportion (%)**


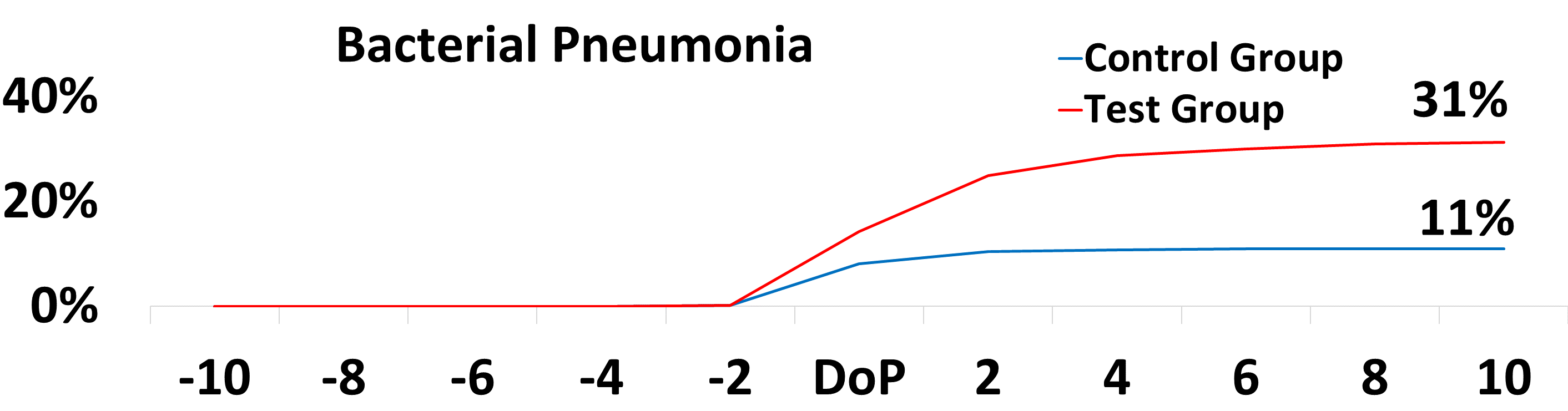

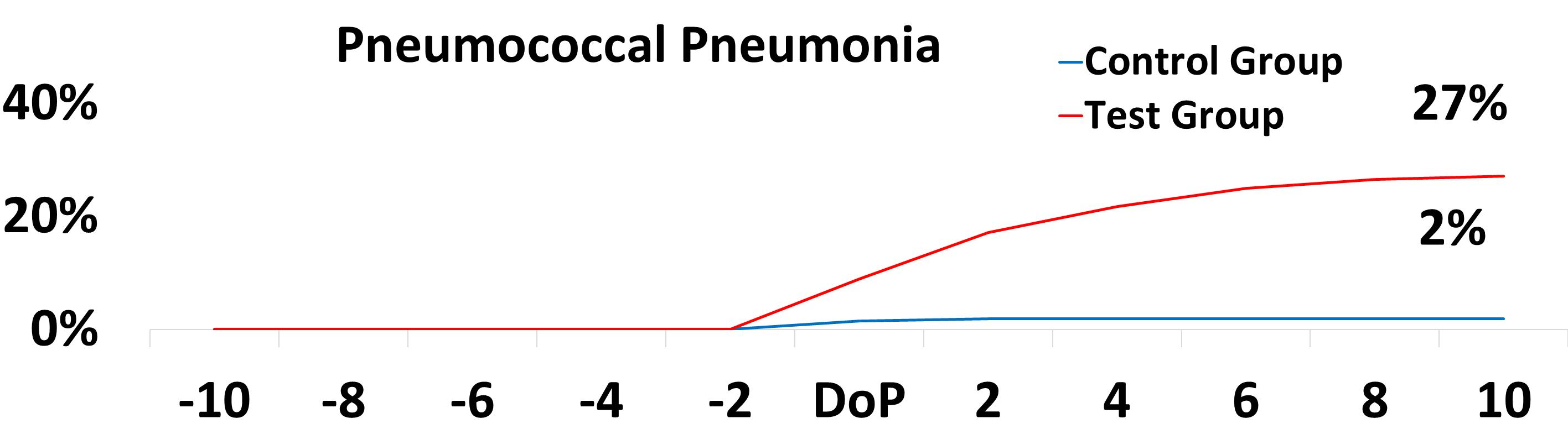


**a)**

**b)**


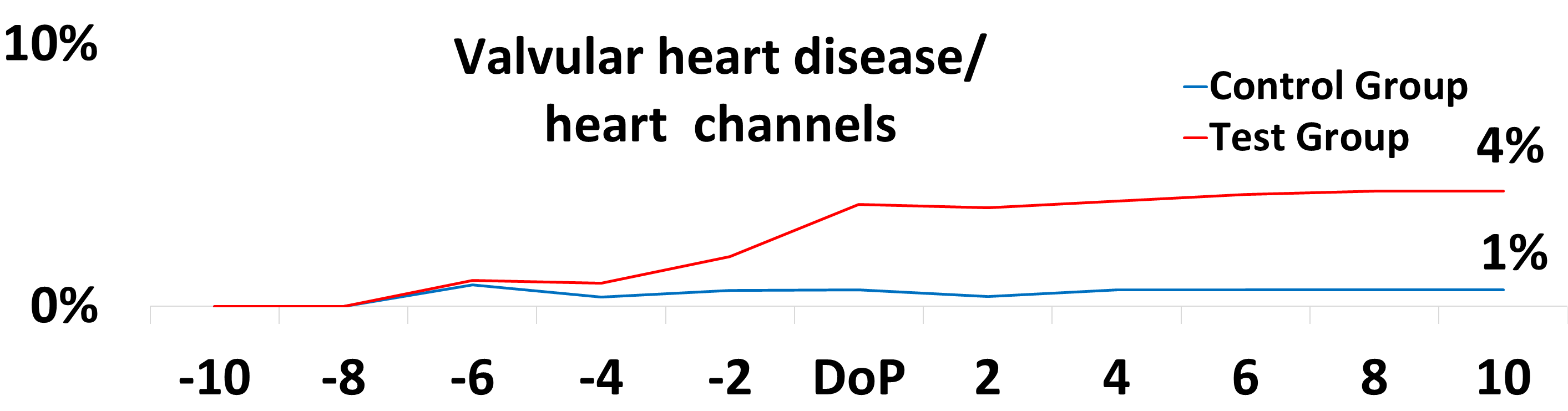


**Years to Pneumonia diagnosis**

**Proportion (%)**

**Years to Pneumonia diagnosis**

**f)**


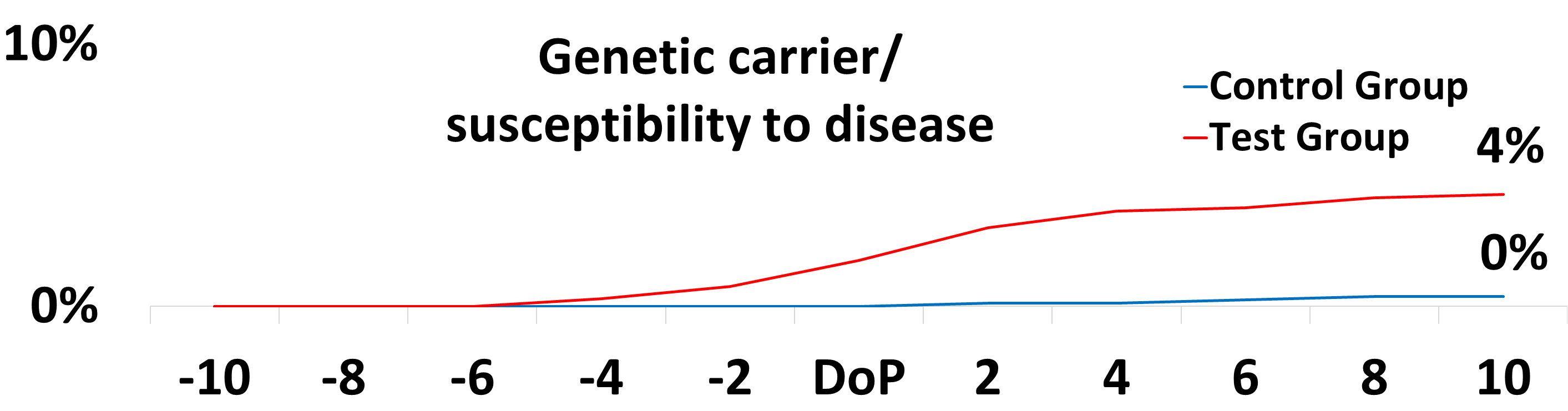

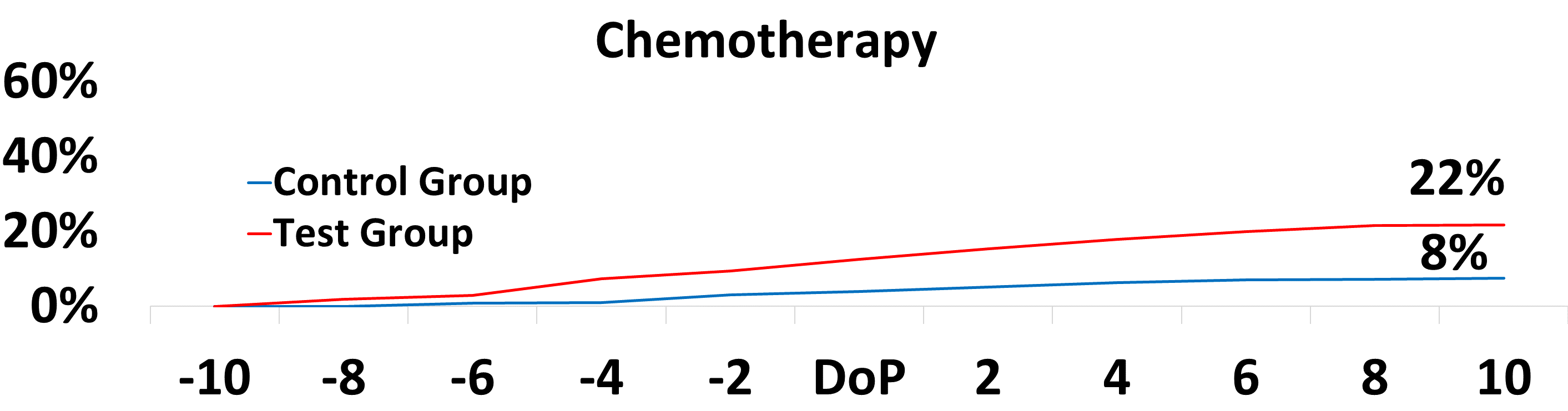


**i)**

**Proportion (%)**

**Years to Pneumonia diagnosis**

**Years to Pneumonia diagnosis**


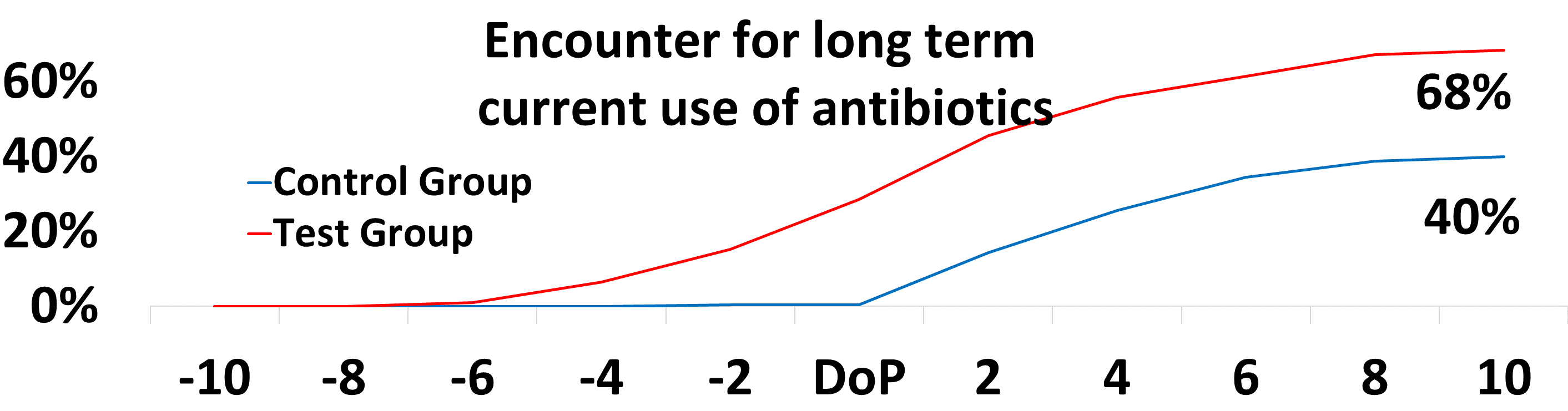


**j)**

**Years to Pneumonia diagnosis**

**Proportion (%)**

**Years to Pneumonia diagnosis**


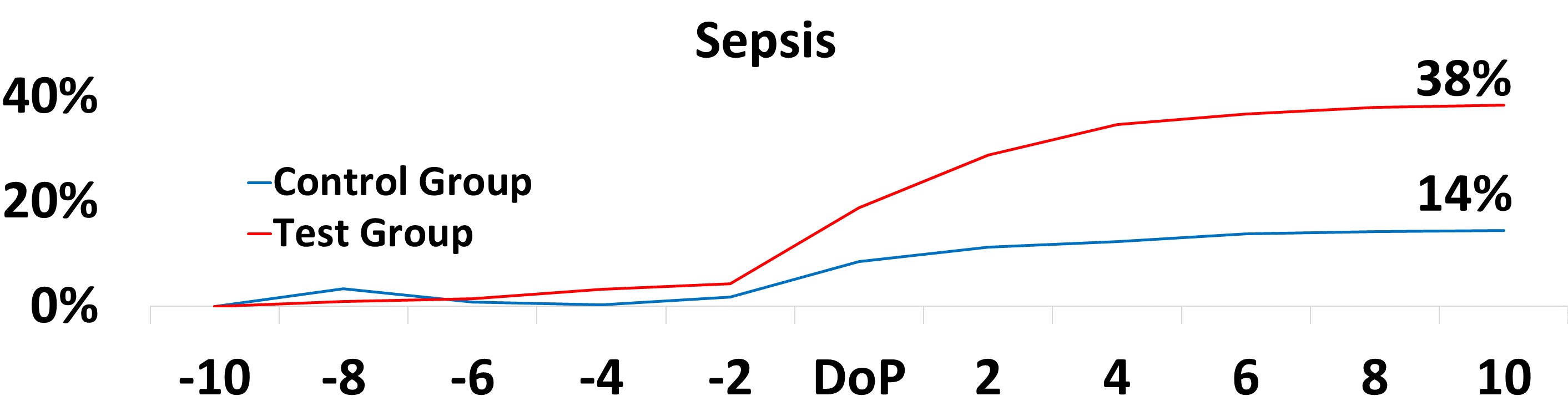

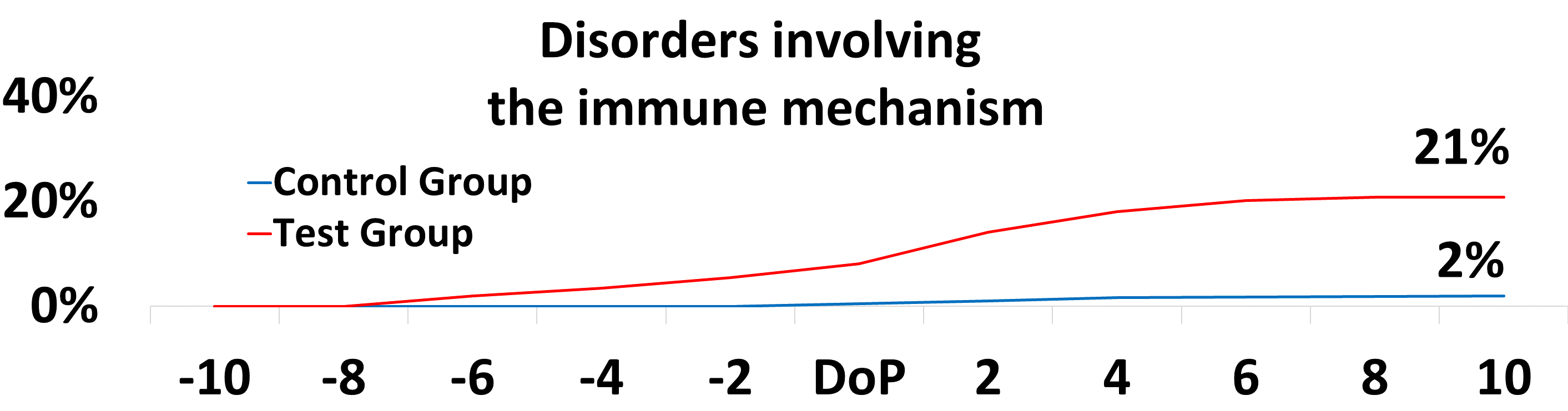


**h)**

**g)**

**Supplementary Figure 9) 20-year time frames of pulmonary (a-d) and non-pulmonary (e-j) clinical phenotypes associated with PI (CID) patients with pneumonia against non-PI patients with pneumonia, prior to and after the diagnosis of pneumonia** (used as a common phenotype between PI cases and controls). To derive fair comparisons, time courses were derived from Cohort 1, in which there was an even number of patients with pneumonia in cases and controls (all 797 in either group).

The illustrations depict the cumulative proportion of patients with each particular phenotype, which equals the sum of the proportions from each of the years preceding or following pneumonia diagnosis. CID: Combined immunodeficiency. The x axis shows years.
