# Supplementary material for: Large-scale deep learning analysis to identify adult patients at risk for combined and common variable immunodeficiencies
Source: Commun Med (Lond). 2023 Dec 20;3:189. doi: 10.1038/s43856-023-00412-8 (PMC10733406; doi:10.1038/s43856-023-00412-8)
Supplement: Supplementary file 2 — Description of Additional Supplementary Files [file 43856_2023_412_MOESM2_ESM.pdf]

Description of Additional Supplementary Files

Supplementary Data 1:

All the ICD-9 codes present in our data were converted to ICD-10 codes using the updated general equivalence mappings (2018 GEMS) from the https://www.cms.gov/ website, provided here.

Supplementary Data 2:

The Short and Long Description, Major and Sub Chapter levels of the PheWAS Phecode v.1.2, as obtained from the most updated ICD Data R package (http://cran.nexr.com/web/packages/icd/icd.pdf, based on which one or more ICD codes were classified into a distinct phenotype.

Supplementary Data 3:

The phenotype mapping file that we created and used based on the PheWAS Phecode v.1.2.

Supplementary Data 4:

We make available 5 main code pieces, which refer to the main data transformation and pre-processing steps up to machine learning model fitting: 1. Code used to convert ICD9 to ICD10 (SQL), 2. Convert ICD to the corresponding disease description (SQL), 3. Convert ICD to phenotypes (SQL), 4. Pivot table with features: ICD disease descriptions (Python) and 5. All machine learning models together with wide and deep, deep only and wide only experimental procedure (Python).

Supplementary Data 5:

A full list of all phenotype ORs, prevalence and statistical significance across cohorts.

Supplementary Data 6:

All pneumonia subtypes identified in our data.
