# Supplementary material for: Large-scale deep learning analysis to identify adult patients at risk for combined and common variable immunodeficiencies
Source: Commun Med (Lond). 2023 Dec 20;3:189. doi: 10.1038/s43856-023-00412-8 (PMC10733406; doi:10.1038/s43856-023-00412-8)
Supplement: Supplementary file 8 — Supplementary Data 6 [file 43856_2023_412_MOESM8_ESM.zip › Supplementary Data 6/Pneumonia subtypes.docx]

**Supplementary Data 6) All pneumonia subtypes identified in our study and their prevalence (%).** Pneumonia prevalence was measured in the largest Cohort 4 which includes all patients extracted in this study (CID+CVID). CID: combined immunodeficiencies, CVID: Common variable immunodeficiencies (see next page).

| **ICD-10** | **Disease description** | **Prevalence (%)** |
| --- | --- | --- |
| J18. 9 | pneumonia unspecified organism | 27.97344 |
| Z87. 01 | personal history of pneumonia recurrent | 10.25357 |
| J15 | bacterial pneumonia not elsewhere classified | 8.397062 |
| J18. 1 | lobar pneumonia unspecified organism | 6.444959 |
| J15. 9 | unspecified bacterial pneumonia | 4.261421 |
| J12 | viral pneumonia not elsewhere classified | 3.094184 |
| J16. 8 | pneumonia due to other infectious organisms not elsewhere classified | 1.921916 |
| J13 | pneumonia due to streptococcus pneumoniae | 1.851479 |
| J15. 1 | pneumonia due to pseudomonas | 1.690481 |
| J15. 6 | pneumonia due to other aerobic gram negative bacteria | 1.549608 |
| J18. 0 | bronchopneumonia unspecified organism | 1.26283 |
| B96. 1 | klebsiella pneumoniae as the cause of diseases classified elsewhere | 1.232642 |
| J12. 89 | other viral pneumonia | 1.091769 |
| J18. 2 | hypostatic pneumonia unspecified organism | 0.890521 |
| J15.212 | pneumonia due to methicillin resistant staphylococcus aureus | 0.88549 |
| J18. 8 | other pneumonia unspecified organism | 0.825116 |
| J11. 00 | influenza due to unidentified influenza virus with unspecified type of pneumonia | 0.628899 |
| J15. 8 | pneumonia due to other specified bacteria | 0.618837 |
| J14 | pneumonia due to hemophilus influenzae | 0.573556 |
| J15. 7 | pneumonia due to mycoplasma pneumoniae | 0.568525 |
| J15. 4 | pneumonia due to other streptococci | 0.528275 |
| B59 | pneumocystosis | 0.523244 |
| J15. 0 | pneumonia due to klebsiella pneumoniae | 0.467901 |
| A40. 3 | sepsis due to streptococcus pneumoniae | 0.467901 |
| J10. 00 | influenza due to other identified influenza virus with unspecified type of pneumonia | 0.407527 |
| J67 | hypersensitivity pneumonitis due to organic dust | 0.397464 |
| J12. 1 | respiratory syncytial virus pneumonia | 0.37734 |
| J85. 1 | abscess of lung with pneumonia | 0.362246 |
| B97. 81 | human metapneumovirus as the cause of diseases classifiedd elsewhere | 0.362246 |
| J67. 9 | hypersensitivity pneumonitis due to unspecified organic dust | 0.347152 |
| B44. 0 | invasive pulmonary aspergillosis | 0.29684 |
| J10. 01 | influenza due to other identified influenza virus with the same other identified influenza virus pneumonia | 0.291809 |
| J84. 116 | cryptogenic organizing pneumonia | 0.286778 |
| J12. 3 | human metapneumovirus pneumonia | 0.266653 |
| B44. 1 | other pulmonary aspergillosis | 0.261622 |
| B37. 1 | pulmonary candidiasis | 0.246528 |
| B06. 81 | rubella pneumonia | 0.226404 |
| J15. 5 | pneumonia due to escherichia coli | 0.216341 |
| J12. 2 | parainfluenza virus pneumonia | 0.201248 |
| J84. 111 | idiopathic interstitial pneumonia not otherwise specified | 0.191185 |
| J84. 2 | lymphoid interstitial pneumonia | 0.150936 |
| P23. 9 | congenital pneumonia | 0.135842 |
| J09. X1 | influenza due to identified novel influenza A virus with pneumonia | 0.135842 |
| J21. 1 | acute bronchiolitis due to human metapneumovirus | 0.130811 |
| J12. 0 | adenoviral pneumonia | 0.130811 |
| J15. 20 | pneumonia due to staphylococcus unspecified | 0.120749 |
| B45. 0 | pulmonary cryptococcosis | 0.120749 |
| J84. 114 | acute interstitial pneumonitis | 0.115717 |
| J20. 0 | acute bronchitis due to mycoplasma pneumoniae | 0.110686 |
| B25. 0 | cytomegaloviral pneumonitis | 0.110686 |
| J15. 3 | pneumonia due to streptococcus group B | 0.110686 |
| B25. 0 | cytomegaloviral pneumonitis | 0.110686 |
