# Supplementary material for: Large-scale deep learning analysis to identify adult patients at risk for combined and common variable immunodeficiencies
Source: Commun Med (Lond). 2023 Dec 20;3:189. doi: 10.1038/s43856-023-00412-8 (PMC10733406; doi:10.1038/s43856-023-00412-8)
Supplement: Supplementary file 9 — Reporting summary [file 43856_2023_412_MOESM9_ESM.pdf]

nature portfolio

Corresponding author(s): Giorgos Papanastasiou

Last updated by author(s): 21/06/23

Reporting Summary

Nature Portfolio wishes to improve the reproducibility of the work that we publish. This form provides structure for consistency and transparency in reporting. For further information on Nature Portfolio policies, see our Editorial Policies and the Editorial Policy Checklist.

Statistics

For all statistical analyses, confirm that the following items are present in the figure legend, table legend, main text, or Methods section.

n/a	Confirmed	
☐	☒	The exact sample size (*n*) for each experimental group/condition, given as a discrete number and unit of measurement
☐	☒	A statement on whether measurements were taken from distinct samples or whether the same sample was measured repeatedly
☐	☒	The statistical test(s) used AND whether they are one- or two-sided<br>*Only common tests should be described solely by name; describe more complex techniques in the Methods section.*
☐	☒	A description of all covariates tested
☒	☐	A description of any assumptions or corrections, such as tests of normality and adjustment for multiple comparisons
☐	☒	A full description of the statistical parameters including central tendency (e.g. means) or other basic estimates (e.g. regression coefficient) AND variation (e.g. standard deviation) or associated estimates of uncertainty (e.g. confidence intervals)
☐	☒	For null hypothesis testing, the test statistic (e.g. *F*, *t*, *r*) with confidence intervals, effect sizes, degrees of freedom and *P* value noted<br>*Give P values as exact values whenever suitable.*
☒	☐	For Bayesian analysis, information on the choice of priors and Markov chain Monte Carlo settings
☒	☐	For hierarchical and complex designs, identification of the appropriate level for tests and full reporting of outcomes
☒	☐	Estimates of effect sizes (e.g. Cohen's *d*, Pearson's *r*), indicating how they were calculated

Our web collection on statistics for biologists contains articles on many of the points above.

Software and code

Policy information about availability of computer code

Data collection	Datasets were sourced from a vendor, i.e., Optum Inc. SQL queries were used to extract and process de-identified data from electronic health records (Optum). The first data extraction step occurs directly inside Dataiku, by extracting data tables i.e., diagnosis and patient data tables, as described in our original manuscript (no SQL code was used for this step). Hierarchical ICD code mapping and ICD code to phenotype conversions were performed using SQL (the "regexp_replace" function, further details described in our Methods section).
Data analysis	All data analyses were performed using custom Python 3.7 scripts and open source libraries (pandas, numpy, scipy, matplotlib, GridSearchCV, scikit-learn for classic machine learning baseline models), and PyTorch widedeep (for deep learning models). Statistical analyses were performed in R (R version 4.1.3). Propensity score matching was performed in R using the MatchIt library. The Optum data used for this study could not be made publicly available due to data use agreements, but can be made available to qualified investigators upon reasonable request. We make available 5 SQL code pieces, which refer to the main data transformation and pre-processing steps up to machine learning model fitting, which we submit as part of our Supplementary material: 1. Code used to convert ICD9 to ICD10 (SQL), 2. Convert ICD to the corresponding disease description (SQL), 3. Convert ICD to phenotypes (SQL), 4. Pivot table with features: ICD disease descriptions (Python) and 5. All machine learning models together with wide and deep, deep only and wide only experimental procedure (Python). Moreover, we give access to the 2018 GEMS mapping, to the processed Long, Short, Major and Sub Chapter descriptions of ICD codes and to the phenotype mapping file that we created and used. We also informed accordingly our code availability statement. Other SQL queries used to perform intermediate data extractions and data content sanity checks cannot be made publicly available, as they are connected with proprietary data. The Python scripts used for training and testing the machine learning (baseline and deep learning) models has been made available.

For manuscripts utilizing custom algorithms or software that are central to the research but not yet described in published literature, software must be made available to editors and reviewers. We strongly encourage code deposition in a community repository (e.g. GitHub). See the Nature Portfolio guidelines for submitting code & software for further information.

Data

Policy information about availability of data

All manuscripts must include a data availability statement. This statement should provide the following information, where applicable:

- Accession codes, unique identifiers, or web links for publicly available datasets
- A description of any restrictions on data availability
- For clinical datasets or third party data, please ensure that the statement adheres to our policy

The Optum data used for this study could not be made publicly available due to data use agreements, but can be made available to qualified investigators upon reasonable request, as stated in the data availability statement. Raw patient-level data could not be made available to reviewers due to legal requirements from Optum, but additional processed data were made available to reviewers in response to their comments and suggestions.

Human research participants

Policy information about studies involving human research participants and Sex and Gender in Research.

Reporting on sex and gender	Data in both PID cases and controls included information from both males and females. PID cases and controls were matched for a number of parameters including their sex, which is reported by primary care practitioners in administrative medical claims. The above information is explicitly described in the Methods section of the manuscript.
Population characteristics	See full details of population characteristics (age and gender, ethnicity and patient history) in Table 1.
Recruitment	US nationally representative medical claims (ICD codes) were used to develop four different PID cohorts (Cohorts 1-4). As discussed in our study limitations, clinical history might be misinformed because of differences across regional, institutional, or individual ICD coding processes. By converting into phenotypes, we aimed to minimize any biases from miscoded disease subtypes. Despite we aimed to diversify our cohort patient characteristics across Cohorts 1-4 to assess disease identification across varied patient population settings, we identified congruent (statistically significant) phenotypes between cohorts which possibly reflect low biases in our data.
Ethics oversight	Optum data were de-identified and received institutional review board approval at the time of linking primary data sources across different US regions (see: https://www.optum.com/content/dam/optum/resources/productSheets/Retrospective-Database-Analysis.pdf). Therefore, a separate ethical approval for this study was not required. In our future work, we aim to re-train our models on Optum data and perform inference on external clinical data, for which we plan to provide a relevant IRB approval.

Note that full information on the approval of the study protocol must also be provided in the manuscript.

Field-specific reporting

Please select the one below that is the best fit for your research. If you are not sure, read the appropriate sections before making your selection.

☒ Life sciences ☐ Behavioural & social sciences ☐ Ecological, evolutionary & environmental sciences

For a reference copy of the document with all sections, see nature.com/documents/nr-reporting-summary-flat.pdf

Life sciences study design

All studies must disclose on these points even when the disclosure is negative.

Sample size

Sample size was automatically derived by including all combined (CID) and common variable immunodeficiency (CVID) patients in the Optum database, which is a nationally representative cohort covering all States. This makes a total of >100 million patient EHRs and medical claims. Hence, the prevalence of CID (2,312 cases) and CVID (19,924 cases) reflect the diagnosed prevalence in the general US population.

A previous study (Mayampurath et al. J Allergy Clin Immunol Pract. 2022 Nov;10(11):3002-3007) assessed a predictive machine learning model by including 247 primary immunodeficiency patients, against 6,422 controls. The moderate performance in this study can mainly be due to the small PID cohort used and the limited clinical history that it was extracted.

Although our sample size in Cohort 3 was larger, it is comparable to another study (Rider et al. PLoS One 16: e0237285) who developed a Bayesian network consisted of known factors, using 3,460 pediatric patients (of whom 50% with PIDs).

Thus, overall our study involved the largest PID cohort that has been assessed in the literature, reflecting the diagnosed CID and CVID prevalence in the general US population.

Data exclusions

We excluded pediatric patients with severe combined immunodeficiency (SCID), as there is a screening program for newborns in USA that

Data exclusions	diagnoses these patients.<br><br>In clinical practice a PID patient may be assigned with multiple ICD codes corresponding to general or subtypes of immunodeficiency terms. To avoid biasing model training, all other (than the PID ICD codes used for defining Cohorts 1-4) immunodeficiency-related features identified were removed as confounding variables. These confounding variables were (with ICD-10 code in parentheses): “other specified immunodeficiencies” (D84.8), “nonfamilial hypogammaglobulinemia” (D80.1), “immunodeficiency with predominantly antibody defects” (D80.9), “other immunodeficiencies” (D84.89), “immunodeficiency unspecified” (D84.9), “selective deficiency of immunoglobulin G [IgG] subclasses” (D80.3), “selective deficiency of immunoglobulin A [IgA]” (D80.2), “selective deficiency of immunoglobulin M [IgM]” (D80.4), “immunodeficiency with predominantly antibody defects unspecified” (D80.9), “antibody deficiency with near-normal immunoglobulins or with hyperimmunoglobulinemia” (D80.6), and “other immunodeficiencies with predominantly antibody defects” (D80.8).
Replication	Reproducible data extraction, cohort generation, pre-processing, and machine learning modeling were created in Python. The Python packages uses raw ICD codes from patients as inputs and identifies primary immunodeficiency patients and statistical metrics, reported in the manuscript.<br><br>The data and metrics reported in the manuscript have been independently reproduced by analytics teams at Pfizer. We replicated the derived machine learning model for primary immunodeficiency patients in four different cohorts:<br>Combined immunodeficiency patients with pneumonia against matched controls with no diagnosis of primary immunodeficiency and pneumonia (discovery cohort; Cohort 1). Note that pneumonia infection is the most frequent severe infection in patients with combined immunodeficiencies. We then replicated the model training by examining if we can identify combined immunodeficiency patients with pneumonia against matched random controls with no diagnosis of primary immunodeficiency with and without pneumonia (Cohort 2). Model training was reproduced to detect combined immunodeficiency patients against matched random controls, both with and without pneumonia (Cohort 3). Finally, to increase further data heterogeneity and re-investigate whether models can accurately identify primary immunodeficiency in diverse patient settings, we aimed to detect combined and common variable immunodeficiency patients against matched random controls, both with and without pneumonia (Cohort 4). Despite these diversified cohorts, we identified congruent statistically significant phenotypes across cohorts.
Randomization	Randomization is not applicable to this study as it is an observational case-control study that used previously collected data. We did randomly select patients for the 80% of the data used for training and 20% used for testing across all datasets and for all models evaluated (TabMLPNet, TabResNet, Logistic Regression, Support Vector Machine).
Blinding	Blinding data analysis mode was applicable throughout this work, because we allowed all clinical history (ICD codes) to be included in the multi-dimensional space for machine learning model training, apart from confounding variables described above (in "Data Exclusions"). Similarly, all ICD codes examined were then converted into phenotypes for statistical analyses reported in the manuscript.

Reporting for specific materials, systems and methods

We require information from authors about some types of materials, experimental systems and methods used in many studies. Here, indicate whether each material, system or method listed is relevant to your study. If you are not sure if a list item applies to your research, read the appropriate section before selecting a response.

Materials & experimental systems

n/a	Involved in the study
☒	☐ Antibodies
☒	☐ Eukaryotic cell lines
☒	☐ Palaeontology and archaeology
☒	☐ Animals and other organisms
☒	☐ Clinical data
☒	☐ Dual use research of concern

Methods

n/a	Involved in the study
☒	☐ ChIP-seq
☒	☐ Flow cytometry
☒	☐ MRI-based neuroimaging
